# Chlorophyll catabolism precedes changes in chloroplast structure and proteome during leaf senescence

**DOI:** 10.1002/pld3.127

**Published:** 2019-03-20

**Authors:** Eyal Tamary, Reinat Nevo, Leah Naveh, Smadar Levin‐Zaidman, Vladimir Kiss, Alon Savidor, Yishai Levin, Yoram Eyal, Ziv Reich, Zach Adam

**Affiliations:** ^1^ The Robert H. Smith Institute of Plant Sciences and Genetics in Agriculture The Hebrew University Rehovot Israel; ^2^ Department of Biomolecular Sciences Weizmann Institute of Science Rehovot Israel; ^3^ Department of Chemical Research Support Weizmann Institute of Science Rehovot Israel; ^4^ de Botton Institute for Protein Profiling The Nancy and Stephen Grand Israel National Center for Personalized Medicine Weizmann Institute of Science Rehovot Israel; ^5^ Institute of Plant Sciences The Volcani Center ARO Rishon LeZion Israel

**Keywords:** Arabidopsis, chlorophyll, chloroplast, photosynthesis, plastoglobule, senescence, thylakoid

## Abstract

The earliest visual changes of leaf senescence occur in the chloroplast as chlorophyll is degraded and photosynthesis declines. Yet, a comprehensive understanding of the sequence of catabolic events occurring in chloroplasts during natural leaf senescence is still missing. Here, we combined confocal and electron microscopy together with proteomics and biochemistry to follow structural and molecular changes during Arabidopsis leaf senescence. We observed that initiation of chlorophyll catabolism precedes other breakdown processes. Chloroplast size, stacking of thylakoids, and efficiency of PSII remain stable until late stages of senescence, whereas the number and size of plastoglobules increase. Unlike catabolic enzymes, whose level increase, the level of most proteins decreases during senescence, and chloroplast proteins are overrepresented among these. However, the rate of their disappearance is variable, mostly uncoordinated and independent of their inherent stability during earlier developmental stages. Unexpectedly, degradation of chlorophyll‐binding proteins lags behind chlorophyll catabolism. Autophagy and vacuole proteins are retained at relatively high levels, highlighting the role of extra‐plastidic degradation processes especially in late stages of senescence. The observation that chlorophyll catabolism precedes all other catabolic events may suggest that this process enables or signals further catabolic processes in chloroplasts.

## 1 INTRODUCTION

The last developmental stage in monocarpic plants is seed production, which is intertwined with leaf senescence. Natural or developmental leaf senescence is autonomously initiated, but can also be triggered by abiotic and biotic stresses such as nutrient deficiency, shading or darkness, or pathogen infection (for reviews, see Guo & Gan, 2005; Gully, Hander, Boller, & Bartels, 2015; Feller, 2016; Liebsch & Keech, 2016). Leaf senescence is accompanied by massive degradation of pigments, proteins, lipids and nucleic acids, whose building blocks are exported to the growing vegetative and reproductive organs of the plant (Lim, Kim, & Nam, 2007; Maillard et al., 2015). The earliest changes during leaf senescence occur in the chloroplast, as photosynthesis declines and mesophyll cells lose their ability to produce carbohydrates. Nuclei and mitochondria remain intact until later stages, allowing expression of senescence‐associated genes (SAGs) and continued energy production while chloroplasts and their content are already being degraded (Gepstein et al., 2003; Inada, Sakai, Kuroiwa, & Kuroiwa, 1998).

The most noticeable aspect of senescence is chlorophyll breakdown, leading to leaf yellowing (Hortensteiner, 2006; Krautler, 2016). Concomitantly, at the microscopic scale, thylakoids are disintegrated and plastoglobules (PGs)—the thylakoid‐associated lipid droplets, increase in number and size (Rottet, Besagni, & Kessler, 2015; van Wijk & Kessler, 2017). The chlorophyll degradation pathway during senescence, involving three different cellular compartments, is now resolved. It starts in the chloroplast by conversion of chlorophyll *b* to chlorophyll *a*, followed by the removal of the Mg ion and the phytol moiety. Next, the cyclic tetrapyrrole is linearized and modified to yield a primary fluorescent chlorophyll catabolite (pFCC), a molecule which is no longer green. The pFCCs are exported from the chloroplast, modified in the cytosol/ER and then transported to the vacuole, where they accumulate as nonfluorescent, nontoxic, chlorophyll catabolites (Kuai, Chen, & Hortensteiner, 2018). As the chlorophyll molecules in the photosynthetic machinery are bound to proteins, chlorophyll breakdown is expected to be linked to protein degradation (Park et al., 2007). However, details on the degradation of chlorophyll‐binding proteins and the involved proteases during senescence are still missing. Possible candidates for this role are proteases of the FtsH and Deg families, which are involved in degradation of photosynthetic proteins during recovery from photoinhibition (Kapri‐Pardes, Naveh, & Adam, 2007; Kato, Sun, Zhang, & Sakamoto, 2012; Kley et al., 2011; Lindahl et al., 2000). The protease FtsH6 has been implicated in the degradation of light‐harvesting antenna proteins (Zelisko, Garcia‐Lorenzo, Jackowski, Jansson, & Funk, 2005), but the claim has been refuted by the same group 6 years later (Wagner et al., 2011). To date, there is no evidence for the involvement of other such proteases. A more likely candidate is the recently discovered plastoglobule protease PGM48, upregulation or downregulation of which advances or delays leaf senescence, respectively (Bhuiyan & van Wijk, 2017).

Evidence for degradation of chloroplast proteins outside the chloroplast has also been accumulating, leading to the current view of multiple pathways delivering chloroplasts or their content for degradation in the central vacuole or the cytosol (Izumi, Ishida, Nakamura, & Hidema, 2017; Michaeli, Honig, Levanony, Peled‐Zehavi, & Galili, 2014; Otegui, 2018). Chlorophagy, a chloroplast‐related form of autophagy (ATG), is responsible for the engulfment of entire photodamaged chloroplasts (Izumi et al., 2017), or of dark‐induced shrunken chloroplasts (Wada et al., 2009) by autophagosomes, and their ultimate fusion with vacuoles where they are degraded by different acid hydrolases. Alternatively, Rubisco‐containing bodies (RCBs), carrying soluble chloroplast proteins (Chiba, Ishida, Nishizawa, Makino, & Mae, 2003; Ishida et al., 2008), also utilize the autophagy machinery to deliver fractions of the chloroplast soluble content into the vacuole. Another form of autophagy‐dependent pathway is that of ATG8‐interacting protein (ATI) that transports thylakoid, stroma and envelope proteins to the vacuole (Michaeli et al., 2014). Aside from these, there are autophagy‐independent pathways. The chloroplast vesiculation (CV) protein is a plastid‐targeted protein that is involved in forming vesicles that eventually are delivered to the vacuole (Wang & Blumwald, 2014). Another type of vesicles that are independent of autophagy is senescence‐associated vacuoles (SAVs) that themselves contain the SAG12 cysteine protease (Carrion et al., 2013; Otegui, 2018), and apparently degrade their cargo in the cytosol. Finally, two ubiquitin E3 ligases, SP1 and PUB4, have been implicated in the ubiquitination of chloroplast outer envelope proteins, resulting in their degradation by the 26S proteasome or delivering them for degradation in vacuoles (Ling, Huang, Baldwin, & Jarvis, 2012; Woodson et al., 2015). Whether all these pathways are involved in dismantling chloroplasts during natural leaf senescence or whether they operate under highly specific conditions, is not clear.

The reported multitude of pathways associated with disintegration of chloroplasts and degradation of their proteins under different physiological conditions prompted us to combine confocal and electron microscopy together with proteomic tools, to obtain a comprehensive view of the changes occurring in chloroplasts during natural leaf senescence in *Arabidopsis thaliana*. We show that chlorophyll catabolism precedes the decrease in photosynthesis capacity, major changes in the thylakoid membranes, and the massive degradation of proteins that ensue during senescence. We also find that, surprisingly, the disappearance of individual chloroplast proteins during senescence is mostly uncoordinated and independent of their inherent stability during earlier developmental stages.

## 2 MATERIAL AND METHODS

### 2.1 Plant material

*Arabidopsis thaliana* plants (Columbia‐0) were grown under short‐day conditions (10 hr light/14 hr dark) at 120 μmol photons m^−2^ s^−1^ at 22°C and 70% humidity for 3 months. Photon flux densities were measured using a LI–250A light meter (LI–COR, USA).

### 2.2 Chlorophyll content

Measurements were performed on intact leaves using a SPAD‐502 meter (Konica‐Minolta, Japan). At least three measurements were performed on each leaf section. Chlorophyll concentrations (nmol chl/cm^2^) were derived according to (Ling, Huang, & Jarvis, 2011).

### 2.3 Sample collection and area calculation

**Figure 1:**
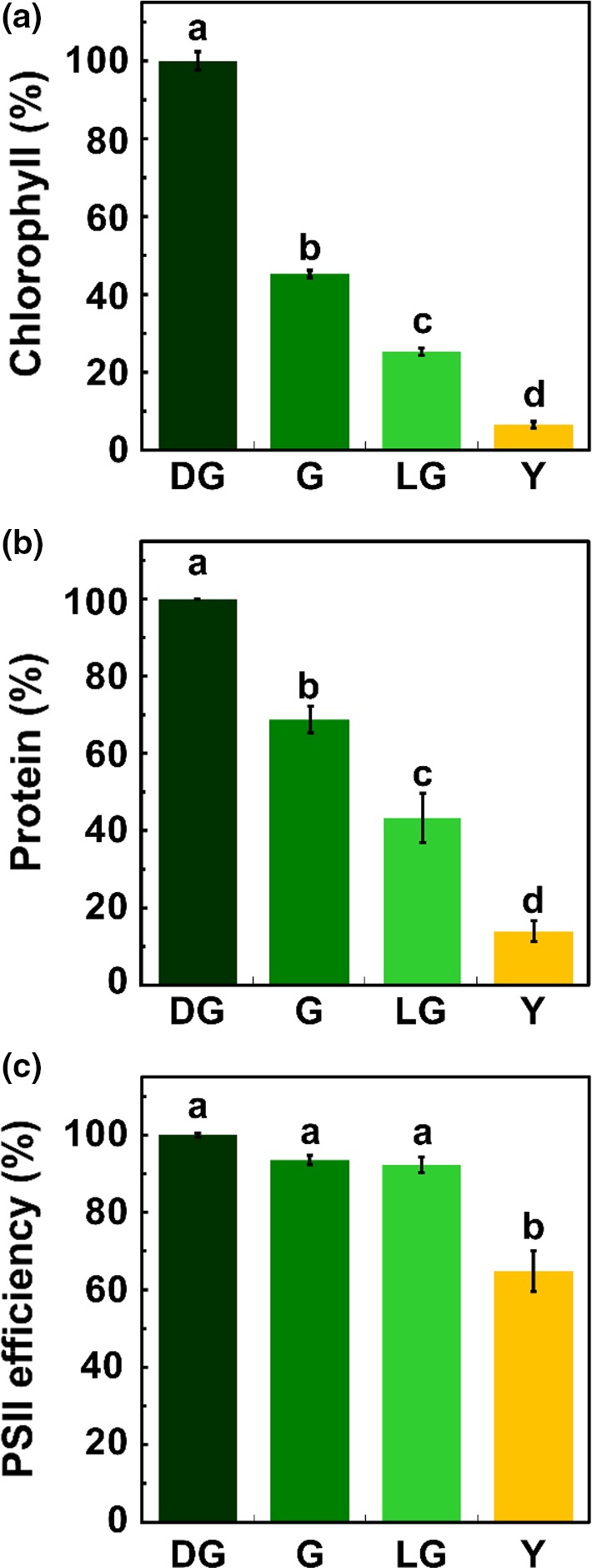
Leaves throughout senescence. General characterization of *Arabidopsis thaliana* leaves throughout four stages of senescence: Dark Green (DG), Green (G), Light Green (LG) and Yellow (Y). (a) Chlorophyll content (*p *<* *0.05, *n *=* *6). 100% = 131.18 nmol chl/cm^2^. (b) Protein levels (*p *<* *0.05, *n *=* *3). 100% = 3.56 μg/mm^2^. (c) PSII photochemical efficiency (Fv/Fm), measured by pulse‐amplitude modulated fluorometry (*p *<* *0.05, *n *=* *4). 100% = 0.76. The data represent mean values, bars shown indicate standard error and letters indicate statistical significance using Tukey‐Kramer HSD test

SPAD measurements were conducted after flowering. Selected sections were dissected and divided into four groups depending on their relative chlorophyll levels (Figure 1): “Dark green” (DG) sections had a dark green color and they constituted the baseline for chlorophyll levels. “Green” (G) leaf sections were segments which have begun de‐greening; their chlorophyll level was ~45% of that of DG leaves. “Light green” (LG) sections were segments in late stages of de‐greening; their chlorophyll level being ~25% of DG leaves. “Yellow” (Y) leaf sections were advanced senescing sections having chlorophyll levels of about 6.5% of those of DG leaves. The areas of the dissected sections were measured using Fiji‐ImageJ and were used as a mean to normalize the data.

### 2.4 Protein samples used for total protein analysis

At least three leaf sections from each stage were pooled to create a sample and triplicates of each stage were used. The samples were frozen in liquid nitrogen and ground while frozen using a pestle and mortar. Protein samples were normalized to leaf area. Sample buffer (0.5 M Tris‐HCl, pH 6.8, 10% [v/v] glycerol, 8% [w/v] SDS, 5 M urea and 20% [v/v] β‐mercaptoethanol) was added based on leaf area (1 μl/mm^2^). Plant protease inhibitors (Protease Inhibitor Cocktail for plant cell and tissue extracts, P9599, Sigma) were also added to the samples according to the volume of the sample, as specified in the product's datasheet.

Samples of the four leaf‐section types (DG, G, LG and Y), normalized to area, were run along with known concentrations of BSA (Albumin from bovine serum, A7906, Sigma) using polyacrylamide gel electrophoresis. The samples were run just until they entered the lower gel in order to prevent the sample from separating, keeping all proteins present as a single band. The gel was then stained with PageBlue Protein Staining Solution (Thermo Scientific, Lithuania) and imaged using LAS 500 (GE Healthcare Bio‐Sciences, Sweden). The stained gel bands were analyzed using Fiji‐ImageJ. A trend line equation of the known concentrations of BSA samples was generated using Excel. Using this equation, the total protein concentration of each sample, present in the single band, was calculated. Three biological replicates were analyzed for each stage.

### 2.5 Fv/Fm determination

Fv/Fm values were determined on detached leaves using an imaging PAM MAXI fluorometer (Heinz Walz, Germany). Prior to measurement, the leaves were dark adapted for 20 min.

### 2.6 Confocal microscopy

Freshly cut leaf samples were imaged using an Olympus FluoView FV1000 IX81 confocal laser‐scanning microscope. Images were acquired with an X60 1.35 NA oil‐immersion objective. To derive the volume, 83, 0.5‐μm‐thick optical sections were collected for each sample. A correction for the distortion along the *z*‐axis of the image was implemented as described previously (Swid et al., 2018). Chlorophyll was excited at 442 nm and the emitted fluorescence was collected using a 655–755 nm bandpass filter.

Three‐dimensional reconstruction and image analysis were carried out using the Imaris software (Bitplane, Switzerland) package. For the analysis of chloroplast volume, the surfaces of chloroplasts were rendered by manual thresholding, following smoothing (using Gaussian filter [~0.4 μm]), and background subtraction (~8 μm, local contrast).

### 2.7 Electron microscopy

Dissected leaves were fixed with 3% paraformaldehyde, 2% glutaraldehyde in 0.1 M cacodylate buffer containing 5 mM CaCl_2_ (pH 7.4). They were then postfixed in 1% osmium tetroxide supplemented with 0.5% potassium hexacyanoferrate trihydrate and potasssium dichromate in 0.1 M cacodylate (1 hr), stained with 2% uranyl acetate in water (1 hr), dehydrated in graded ethanol solutions and embedded in Agar 100 epoxy resin (Agar Scientific Ltd., Stansted, UK). Ultrathin sections (70–90 nm) were viewed and photographed with a FEI Tecnai SPIRIT (FEI, Eidhoven, Netherlands) transmission electron microscope operating at 120 kV and equipped with an EAGLE CCD Camera. Measurements of chloroplasts, starch bodies, and plastoglobules were carried out using Fiji (ImageJ).

### 2.8 Mass spectrometry

Mass spectrometry was performed at the De Botton Protein Profiling institute of the Nancy and Stephen Grand Israel National Center for Personalized Medicine, Weizmann Institute of Science, essentially as recently described (Butenko et al., 2018). Samples were subjected to in‐solution tryptic digestion using a modified Filter Aided Sample Preparation protocol (FASP). All chemicals were from Sigma Aldrich, unless stated otherwise. Sodium dodecyl sulfate buffer (SDT) included: 4% (w/v) SDS, 100 mM Tris‐HCl pH 7.6, 0.1 M DTT. Urea buffer (UB): 8 M urea (Sigma, U5128) in 0.1 M Tris‐HCl pH 8.0, UA: 8 M urea in 0.1 M Tris‐HCl, pH 8.0, 50 mM ammonium bicarbonate. Cells were dissolved in 100 μl SDT buffer and lysed for 3 min at 95°C, then spun down at 16,000 RCF for 10 min, 30 μl was mixed with 200 μl UB and loaded onto 30 kDa molecular weight cutoff filters and spun down. Two hundred‐microliters of UA was added to the filter unit and centrifuged at 14,000 *g* for 40 min. Trypsin was then added and samples were incubated at 37°C overnight. Digested proteins were then spun down, acidified with trifloroacetic acid and stored in −80°C until analysis.

ULC/MS grade solvents were used for all chromatographic steps. Each sample was fractionated using high pH reversed phase followed by low pH reversed phase separation. Two hundred‐micrograms of digested protein was loaded using high Performance Liquid Chromatography (Agilent 1260 uHPLC). Mobile phase was: (a) 20 mM ammonium formate pH 10.0, (b) acetonitrile. Peptides were separated on an XBridge C18 column (3 × 100 mm, Waters) using the following gradient: 3% B for 2 min, linear gradient to 40% B in 50 min, 5 min to 95% B, maintained at 95% B for 5 min and then back to initial conditions. Peptides were fractionated into 15 fractions. The fractions were then pooled: 1 with 8, 2 with 9, 3 with 10, 4 with 11, 5 with 12, 6 with 13 and 7 with 14–15. Each fraction was dried in a SpeedVac, then reconstituted in 25 μl in 97:3 acetonitrile: water + 0.1% formic acid. Each pooled fraction was then loaded using split‐less nano‐Ultra Performance Liquid Chromatography (10 kpsi nanoAcquity; Waters, Milford, MA, USA). The mobile phase was: (a) H_2_O + 0.1% formic acid and (b) acetonitrile + 0.1% formic acid. Desalting of the samples was performed online using a reversed‐phase C18 trapping column (180 μm internal diameter, 20 mm length, 5 μm particle size; Waters). The peptides were then separated using a T3 HSS nano‐column (75 μm internal diameter, 250 mm length, 1.8 μm particle size; Waters) at 0.35 μl/min. Peptides were eluted from the column into the mass spectrometer using the following gradient: 4% to 35% B in 150 min, 35% to 90% B in 5 min, maintained at 95% for 5 min and then back to initial conditions.

The nanoUPLC was coupled online through a nanoESI emitter (10 μm tip; New Objective; Woburn, MA, USA) to a quadrupole orbitrap mass spectrometer (Q Exactive Plus, Thermo Scientific) using a FlexIon nanospray apparatus (Proxeon). Data were acquired in data‐dependent acquisition (DDA) mode, using a Top20 method. MS1 resolution was set to 60,000 (at 400 *m*/*z*) and maximum injection time was set to 20 ms. MS2 resolution was set to 17,500 and maximum injection time of 60 ms.

### 2.9 Data processing

Raw data were imported into the Expressionist^®^ software (Genedata) and processed as previously described (Shalit, Elinger, Savidor, Gabashvili, & Levin, 2015). The software was used for retention time alignment and peak detection of precursor peptides. A master peak list was generated from all MS/MS events and sent for database searching using Mascot v2.5 (Matrix Sciences). Data were searched against the *Arabidopsis thaliana* protein database (http://www.uniprot.org/) appended with 125 common laboratory contaminant proteins. Fixed modification was set to carbamidomethylation of cysteines and variable modification was set to oxidation of methionines. Search results were then filtered using the PeptideProphet2 algorithm to achieve maximum false discovery rate of 1% at the protein level. Peptide identifications were imported back to Expressions to annotate identified peaks. Quantification of proteins from the peptide data was performed using a published script (Shalit et al., 2015). Data were normalized based on the total ion current. Protein abundance was obtained by summing the three most intense, unique peptides per protein.

### 2.10 Statistical and bioinformatic analyses

#### 2.10.1 Morphometric analysis

Differences in chloroplast and cell volumes, as well as in the areas of chloroplasts and starch granules, and in the area and number of plastoglobules were tested using one‐way ANOVA. Comparison of all pairs was performed using the Tukey‐Kramer HSD test. All statistical tests were performed using JMP PRO (SAS, USA).

#### 2.10.2 Proteomic analysis

Analyses were performed with Perseus software (version 1.5.6.0) (Tyanova et al., 2016). Categorical annotations were supplied in the form of Gene Ontology (GO) “Biological Process”, “Molecular Function”, and “Cellular Component” types. All annotations were obtained from the UniProt database (www.uniprot.org). Minimum valid values per protein were seven (out of twelve) samples. Missing values were replaced from normal distribution. Multiple sample test ANOVA with 0.05 FDR and 250 randomizations were performed and proteins that passed this test were clustered, after z‐scoring, using hierarchical clustering.

Proteomaps were generated using the Proteomaps 2.0 site (http://bionic-vis.biologie.uni-greifswald.de/). Protein lists uploaded to create the proteomaps were based on proteins present in each stage which passed the multiple sample test ANOVA with 0.05 FDR. Up and downregulated protein groups were based on the hierarchical clustering groups generated using Perseus.

Proteins were clustered according to the change in their levels based on hierarchical clustering obtained using Perseus (Tyanova et al., 2016). Overrepresentation of GO terms was determined using the Panther overrepresentation test (http://pantherdb.org) (Mi et al., 2017). The base group used for this analysis consisted of all proteins detected in the MS analyses, which had a minimum of seven valid values per protein. After the replacement of missing values, 6,034 out of 6,076 proteins passed this screening and served as the base group. Only results with a *p*‐value < 0.05 were used. For the manual protein group assembly, only proteins with a minimum of two peptide identifications and whose level changed by more than 1.5‐folds were used.

## 3 RESULTS

### 3.1 Chlorophyll levels decrease faster than protein during natural senescence

Although dark‐induced senescence is easier to study, as it provides a very clear reference point (transfer to darkness), the accumulating data suggest that it is different in many respects from natural senescence (Buchanan‐Wollaston et al., 2005) and therefore cannot serve as a reliable model for natural senescence. We thus chose to concentrate on the latter. As yellowing is the most distinct visual phenotype associated with natural leaf senescence, we decided to use leaf color as the basis for comparing different stages of this process. Arabidopsis plants were grown in short‐day photoperiod under optimal conditions for 3 months, before dissecting their leaves and classifying them according to their color: dark green (DG), green (G), light green (LG) and yellow (Y). Figure 1a shows the relative level of chlorophyll in each developmental group, ranging from ca. 130 nmol/cm^2^ to <10 nmol/cm^2^. Protein levels also decreased in these leaves, but at a slower rate (Figure 1b). For instance, whereas upon the transition from DG to G the leaves lost more than 50% of their chlorophyll, they did retain about 70% of their protein content. Likewise, LG leaves contained ca. 25% of their initial chlorophyll but still possessed more than 40% of their proteins. To gain insight into the photosynthetic capacity of the leaves at the different developmental stages, photosystem II (PSII) maximum quantum yield (Fv/Fm) was determined using PAM fluorometry. A significant change was observed only upon the transition from LG to Y, when Fv/Fm dropped from more than 0.7 to less than 0.5 (Figure 1c). Taken together, it is evident that chlorophyll catabolism proceeds faster than the decrease in PSII efficiency.

### 3.2 Although chlorophyll levels drop in half, chloroplast size remains constant during early stages of senescence

**Figure 2:**
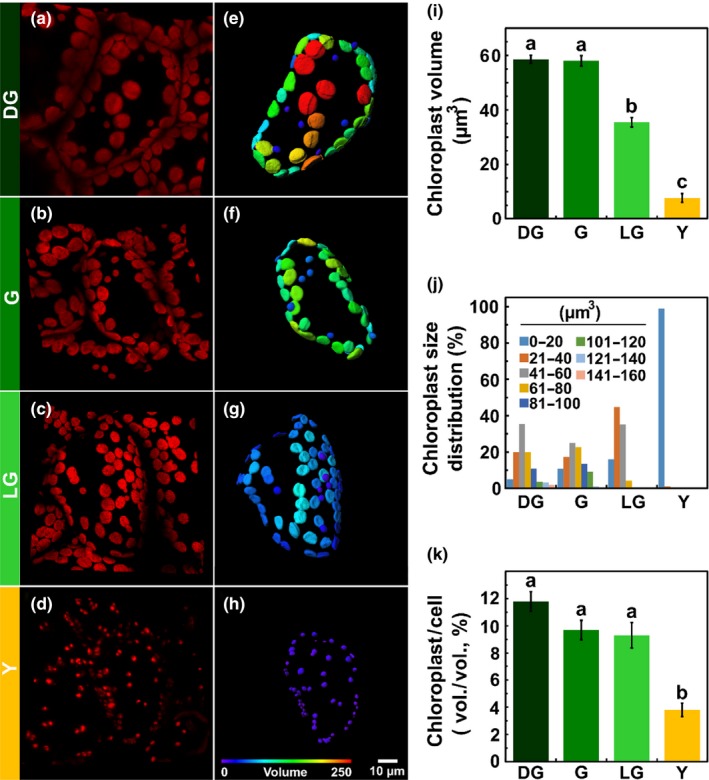
Chloroplasts during leaf senescence. Characterization of chloroplasts from live tissues of *Arabidopsis thaliana* leaves, using confocal imaging, throughout four stages of leaf senescence: Dark Green (DG), Green (G), Light Green (LG) and Yellow (Y). (a–d) Three‐dimensional reconstructed confocal images of mesophyll cells. All images are of the same magnification. (e–h) Three‐dimensional model of the chloroplasts present in a cell shown in panels (a)–(d), respectively. Coloring is based on volume. (i) Chloroplast volume. (j) Volume distribution, calculated from the 3D models. (*p *<* *0.05, *n *≥* *128). (k) Total chloroplast volume out of the cell volume, calculated from the 3D models (*p *<* *0.05, *n *≥* *25). The data represent mean values, bars indicate standard error and letters indicate significance using Tukey‐Kramer HSD test

To monitor changes in the size of chloroplasts throughout senescence, 3D reconstructed confocal images were obtained (Figure 2a–d). Differential coloring of these images, based on chloroplasts’ size, is presented in Figure 2e–h. Interestingly, although chlorophyll levels dropped by more than half during the transition from DG to G, the average volume of the chloroplasts remained constant, at approximately 60 μm^3^. Thereafter, it decreased to 35 and 8 μm^3^ in the LG and Y stages, respectively (Figure 2i). The distribution of chloroplast volumes within each stage also changed during senescence. In DG and G leaves, a wide range of chloroplast sizes, ranging in volume from 10 to 154 μm^3^, was observed (Figure 2j). Concomitant with the progression of senescence, the fraction of smaller chloroplasts increased, up to Y leaves where almost all chloroplasts had a volume smaller than 20 μm^3^. The 3D confocal reconstructions also allowed estimating the fraction of cell volume occupied by chloroplasts at the different developmental stages. As shown in Figure 2k, throughout the transition from DG to LG, this fraction does not change significantly, amounting to about 0.1. In Y leaves, however, this fraction drops by more than twofold to about 0.04.

### 3.3 Ultrastructural changes in chloroplasts during senescence

**Figure 3:**
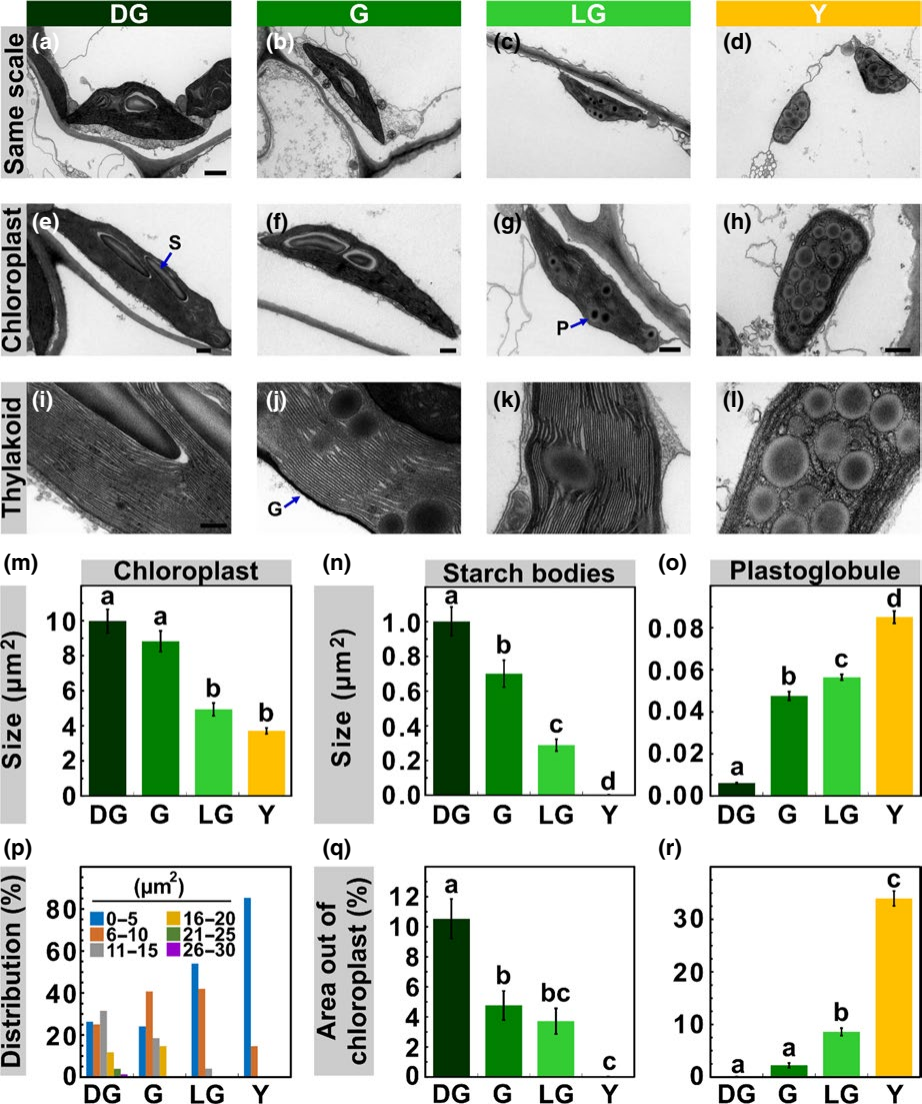
Chloroplast Structure Throughout Senescence. (a‐h) Transmission electron microscopy (TEM) images of chloroplasts present in mesophyll cells of *Arabidopsis thaliana* during stages DG, G, LG and Y. Scale bars: a–d, 1 μm, e–h, 500 nm. (i–l) TEM images of thylakoids. Scale bar, 200 nm. Average chloroplast area (m) and size distribution (p), calculated from TEM images (*p *<* *0.05, *n* ≥ 50). (n) Starch bodies average size and (q), their percentage out of the chloroplast total area, as calculated from TEM images (*p *<* *0.05, *n* ≥ 40). (o) Plastoglobules average size and (r), their percentage out of the chloroplast total area (*p *<* *0.05, *n* ≥ 30). The data represent mean values, bars shown indicate standard error and letters indicate significance

Transmission electron microscopy (TEM) of chemically fixed leaf samples from the four developmental stages revealed the ultrastructural changes within chloroplasts as they senesce. Most noticeable are the decrease in the size of chloroplasts, disappearance of starch bodies, increase in number and size of plastoglobules (PGs), and disintegration of the thylakoid membranes (Figure 3a–l). Average chloroplast area reaches half of its original value (of ca. 10 μm^2^) only at the LG stage (Figure 3m), similar to the trend observed in the confocal images (Figure 2i). However, the TEM images reveal no further significant changes in the area of the chloroplasts upon transition to the Y stage, in contrast to the confocal 3D reconstructions, which indicate an additional decrease in chloroplast volume following this transition. This difference was found to be due to asymmetric shrinking of chloroplasts during the transition from LG to Y. Specifically, the shrinkage along the polar axis (*Z*) was half of that along the *XY* plane. Since the TEM is only 2D and does not include one of the equatorial axes, the change in area between the stages is much less pronounced than the change in volume calculated from 3D confocal microscopy. As was observed in the confocal images, the TEM analysis reveals a narrower distribution of chloroplast sizes, shifted toward smaller values, as senescence progresses (Figures 2j and 3p).

Starch granules are expectedly diminished during senescence, decreasing in size early on during the process (Figure 3n,q). In contrast, PGs dramatically increase in size during senescence (Figure 3o). Whereas PGs in DG chloroplasts have an average size of 0.006 μm^2^, they grow more than 10‐fold to 0.085 μm^2^ in Y chloroplasts, where they occupy about one‐third of the chloroplast area (Figure 3r).

Surprisingly, the thylakoid membranes, which accommodate almost all of the chlorophyll in the cell, retain their normal appearance until relatively late into senescence, namely the LG stage, at which 75% of the chlorophyll is already degraded. Thylakoid networks are almost indistinguishable, both in ultrastructure and overall organization, in DG, G and LG leaves (Figure 3i–k). In sharp contrast, in Y chloroplasts, which are filled with PGs, only a few swelled and wrinkled thylakoids, exhibiting only little and occasional stacking, are observed (Figure 3l).

### 3.4 Global changes in leaf proteome during natural senescence

**Figure 4:**
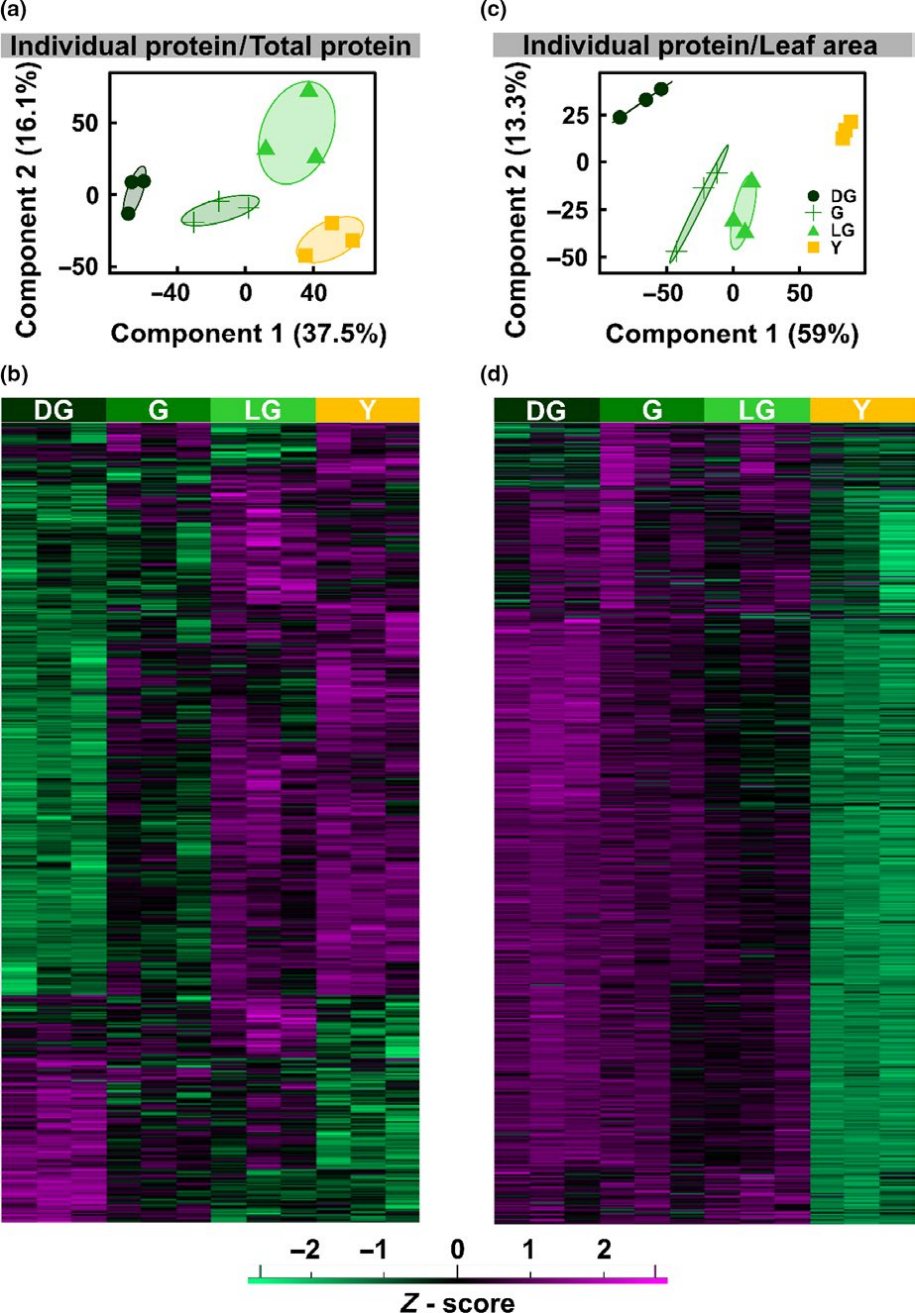
PCA and Hierarchical Clustering of Protein MS Data. Overview of proteomic data as obtained from protein 2D‐LC‐MS/MS analysis on extracts of *Arabidopsis thaliana* senescing leaf samples during the four stages of senescence. a, c: Principal component analysis (PCA) of the samples, normalized to total protein (a) or leaf area (c). b, d: Hierarchical clustering of the proteins detected, based on equal protein loading (b) or normalized to leaf area (d)

To further characterize the senescence process, label‐free, shotgun proteomics was applied. To maximize the number of analyzed proteins, 2D‐LC‐MS/MS (Delahunty & Yates, 2005) was conducted on samples from the aforementioned four developmental groups. Regardless of the decrease in the total amount of protein per leaf area that accompanies senescence (Figure 1b), equal amounts of protein were subjected to the analysis. In total, the proteomic data (see Supporting Information Dataset S1) inferred to 6,076 proteins, 5,000 of them detected by at least two peptides, allowing their quantification. Out of these proteins, 4,894 were detected in all samples, that is, in three replicates in each of the four developmental stages. Principle component analysis (PCA) shows that samples from the four different developmental stages are well separated from each other (Figure 4a). Hierarchical clustering of the 3,570 proteins that passed the ANOVA test (*p *<* *0.05) revealed that, although the total amount of protein decreased during senescence (Figure 1b), the relative levels (i.e., the proportion of a given protein out of equal amounts of protein injected into the MS) of more than three quarters of the proteins were upregulated (Figure 4b). This upregulation might be due to the decrease in the level of highly abundant proteins such as Rubisco.

When the MS data are normalized to leaf area, the picture obviously changes. Here too, PCA reveals that samples belonging to different stages cluster separately (Figure 4c). However, as can be expected, when normalized, the level of almost all proteins is found to decrease as senescence proceeds (Figure 4d). The kinetics of the decrease are quite heterogeneous, with the levels of some proteins decreasing already at the G stage whereas others begin to decrease only at LG. Regardless of these differences, the levels of almost all proteins are highly reduced in the Y stage.

**Figure 5:**
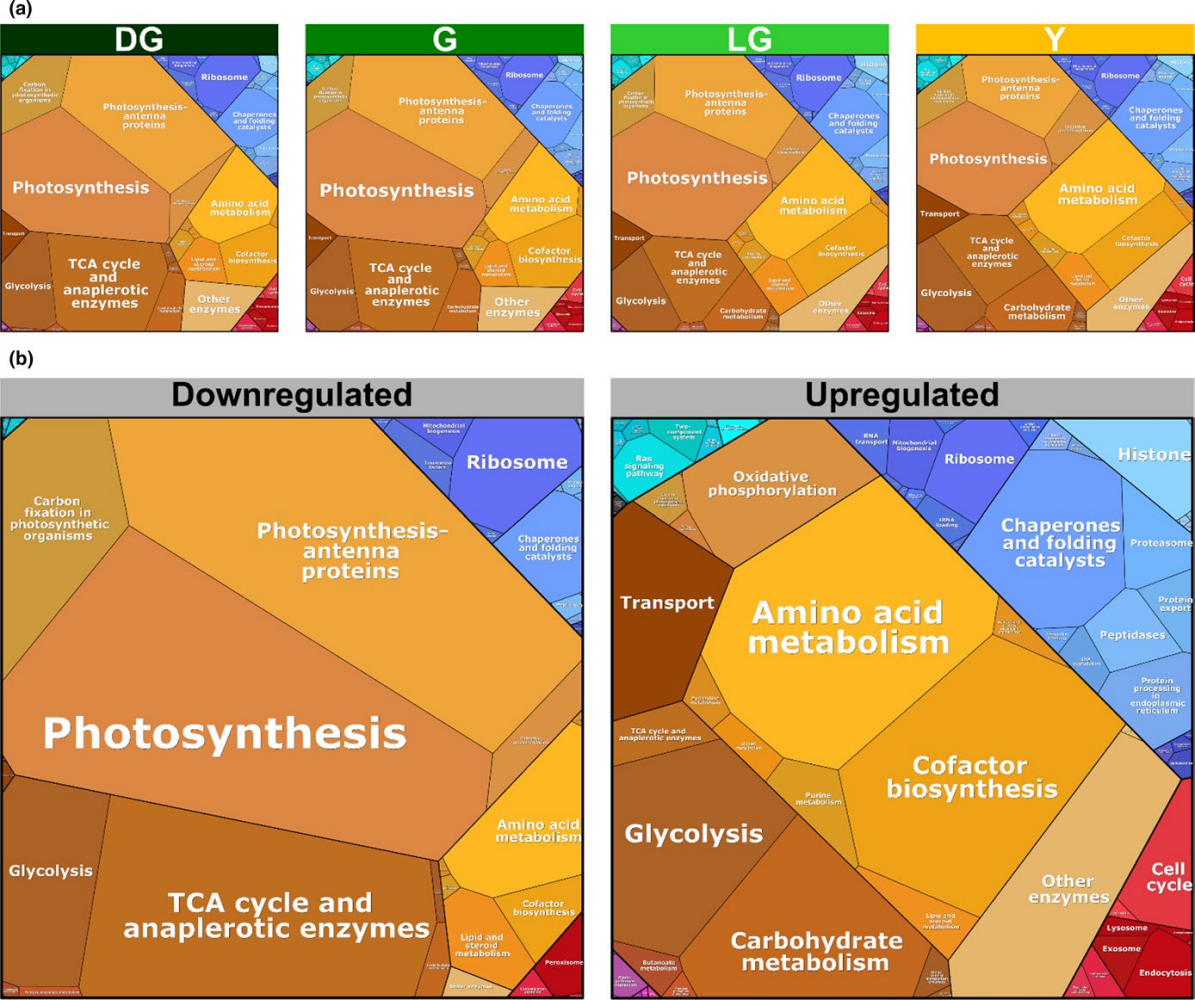
Quantitative composition of proteomes during senescence. Visual representation of proteomic data of *Arabidopsis thaliana* mesophyll cells during four stages of leaf senescence, based on proteomic data normalized to total protein. ~3,500 proteins that passed the ANOVA significance test (*p *<* *0.05) were used for this analysis. (a) Proteomaps of total proteins. (b) Proteomaps of downregulated and upregulated proteins

To visually portray a general scheme of the dynamics between different protein groups during senescence, “proteomaps” (Liebermeister et al., 2014) of the four stages were generated (Figure 5a). Proteomaps represent the abundance of different functional groups of proteins within proteomes. Throughout senescence, subgroups belonging to “Metabolism” (colored yellow and brown) account for most of the protein mass, followed by protein groups classified as “Genetic information processing” (blue), “Cellular processes” (red) and “Environmental information processing” (turquoise) (Figure 5a). Zooming into the “Metabolism” group reveals a subtle, yet continuous change in the ratios between the subgroups during senescence. The major subgroups that decrease are part of “Energy metabolism”, composed mainly of “Photosynthesis” and “Photosynthesis antenna proteins” subgroups, and “Central carbon metabolism”, which consists mainly of “TCA cycle and anaplerotic enzymes” (Figure 5a). The major protein subgroup that is upregulated is “Amino acid metabolism”, which is part of the “Biosynthesis” group (Figure 5a).

The functional categories comprising the population of downregulated proteins clearly differ from those that are upregulated (Figure 5b). We considered here down and upregulated proteins according to the hierarchical clustering presented in Figure 4b, encompassing all stages. In the former, the vast majority is proteins belonging to “Metabolism” followed by “Genetic information processing” and “Cellular Processes”. The major constituents of the first group are “Photosynthesis” and “Photosynthesis antenna” proteins. These are followed by proteins belonging to “TCA cycle and anaplerotic enzymes” and “Carbon fixation in photosynthetic organisms”. Proteins belonging to “Ribosome” and “Chaperone and folding catalysts” make up the largest groups in “Genetic information processing”. Among the proteins that were upregulated in “Metabolism”, most are proteins classified under “Amino acid metabolism” and “Cofactor biosynthesis”. Within the upregulated proteins in “Genetic information processing”, those categorized under “Chaperone and folding catalysts” constitute the largest group (Figure 5b).

### 3.5 Downregulated proteins are enriched in chloroplast‐related processes

To unravel groups enriched within proteins that are downregulated during senescence, we used Gene Ontology (GO) term enrichment analysis. Almost all of the groups detected were linked to chloroplasts (Table 1, upper part). Chlorophyll and starch biosynthesis were the two leading biological processes (BP) overrepresented in downregulated proteins, with enrichment factors of 4.54 and 4.04, respectively. This is in accordance with the decrease in chlorophyll levels and disappearance of starch granules during senescence. Photosynthesis was the next overrepresented BP with an enrichment factor of 2.58. The last overrepresented BP was response to cytokinins, with a similar enrichment factor. Cytokinins act to delay senescence and their levels drop during this process (Zwack & Rashotte, 2013). Another group that is related to cytokinins is a molecular function (MF) group—“Phosphorelay response regulatory activity”, composed of type B Arabidopsis response regulator (ARR) proteins, which act in the cytokinin signaling pathway (Hill et al., 2013). Notably, all cell compartment (CC) groups overrepresented in downregulated proteins are located within the chloroplast, with enrichment factors ranging from 2.11 to 4.04 (Table 1, upper part).

**Table 1:** Overrepresentation of protein groups in downregulated and upregulated proteins

GO type^a^	Group	Enrichment factor^b^	*p*‐Value^c^
Downregulated (1015)^d^			
MF	Phosphorelay Response Regulator Activity (GO:0000156)	5.91	3.61E−03
BP	Chlorophyll Biosynthetic Process (GO:0015995)	4.54	5.11E−05
CC	Photosystem I (GO:0009522)	4.04	1.00E−02
BP	Starch Biosynthetic Process (GO:0019252)	4.04	3.83E−02
CC	Stromule (GO:0010319)	3.73	4.18E−02
CC	Chloroplast Thylakoid Membrane (GO:0009535)	2.72	3.81E−15
BP	Photosynthesis (GO:0015979)	2.58	2.11E−05
BP	Response to Cytokinin (GO:0009735)	2.54	2.35E−05
CC	Chloroplast Stroma (GO:0009570)	2.33	3.22E−22
CC	Chloroplast Membrane (GO:0031969)	2.11	1.18E−02
Downregulated at G (241)			
BP	Starch Biosynthetic Process (GO:0019252	10.01	9.61E−03
BP	Chlorophyll Biosynthetic Process (GO:0015995)	7.48	1.76E−02
Downregulated at LG (60)			
BP	Photosystem II Assembly (GO:0010207)	37.49	5.18E−03
CC	Photosystem II Oxygen Evolving Complex (GO:0009654)	28.12	4.22E−03
BP	Photosynthesis, Light Harvesting (GO:0009765)	22.50	3.79E−02
Upregulated (2556)			
BP	Defense Response to Other Organism (GO:0098542)	1.41	3.89E−02
BP	Cellular Catabolic Process (GO:0044248)	1.36	1.39E−02
CC	Cell Wall (GO:0005618)	1.35	2.04E−02
CC	Extracellular Region (GO:0005576)	1.35	2.73E−05
CC	Vacuole (GO:0005773)	1.32	1.35E−03
CC	Plasma Membrane (GO:0005886)	1.23	2.42E−04
CC	Endomembrane System (GO:0012505)	1.22	6.82E−03
BP	Defense Response to Other Organism (GO:0098542)	1.41	3.89E−02
BP	Cellular Catabolic Process (GO:0044248)	1.36	1.39E−02
CC	Cell Wall (GO:0005618)	1.35	2.04E−02
CC	Extracellular Region (GO:0005576)	1.35	2.73E−05

a GO types: Cell Compartment (CC), Biological Process (BP), and Molecular Function (MF).

b As calculated by the Perseus software platform. Enrichment factor = [(Intersection size)·(Total size)]/[(Selection size)·(Category size)].

c Proteins that passed the ANOVA significance test (*p *<* *0.05).

d Numbers in brackets indicate the number of proteins in the analyzed group.

**Figure 6:**
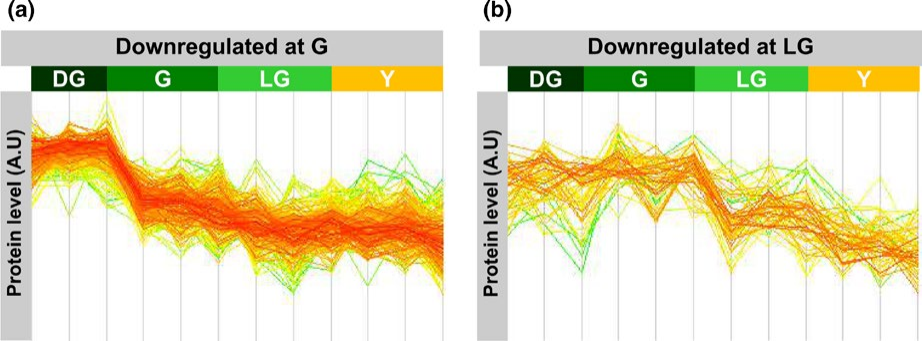
Different patterns of protein downregulation. Downregulated proteins were divided based on the pattern of the change in their level. (a) proteins downregulated early in senescence, after the DG stage. (b) proteins downregulated upon the transition to the LG stage

Downregulated proteins were further divided into different groups according to their kinetics of decline. As shown in Figure 6, the level of some proteins starts to decline upon the transition from DG to G, whereas others start declining later on, during the transition from G to LG. Starch and chlorophyll biosynthesis were the two BP protein groups displaying the former pattern, whereas proteins related to PSII composition and assembly, as well as its peripheral antenna complement began declining later on (Table 1).

Notably, none of the upregulated enriched groups was related to chloroplasts. Bioprocesses enriched here were “Defense response to other organisms” and “Cellular catabolic processes”, with enrichment factors of 1.41 and 1.36, respectively (Table 1). Cell compartments overrepresented were the cell wall, extracellular region, vacuole, plasma membrane and endomembranes.

### 3.6 Behavior of specific chloroplast proteins during senescence

GO terms help to quickly categorize proteins into groups. However, the vast majority of GO terms is based on automatic predictions (Huntley, Sawford, Martin, & O'Donovan, 2014) and, hence, may not include all proteins of a certain group or include more proteins than necessarily belonging to the group. We therefore manually grouped proteins based on known pathways or complexes and normalized their expression levels to leaf area.

**Figure 7:**
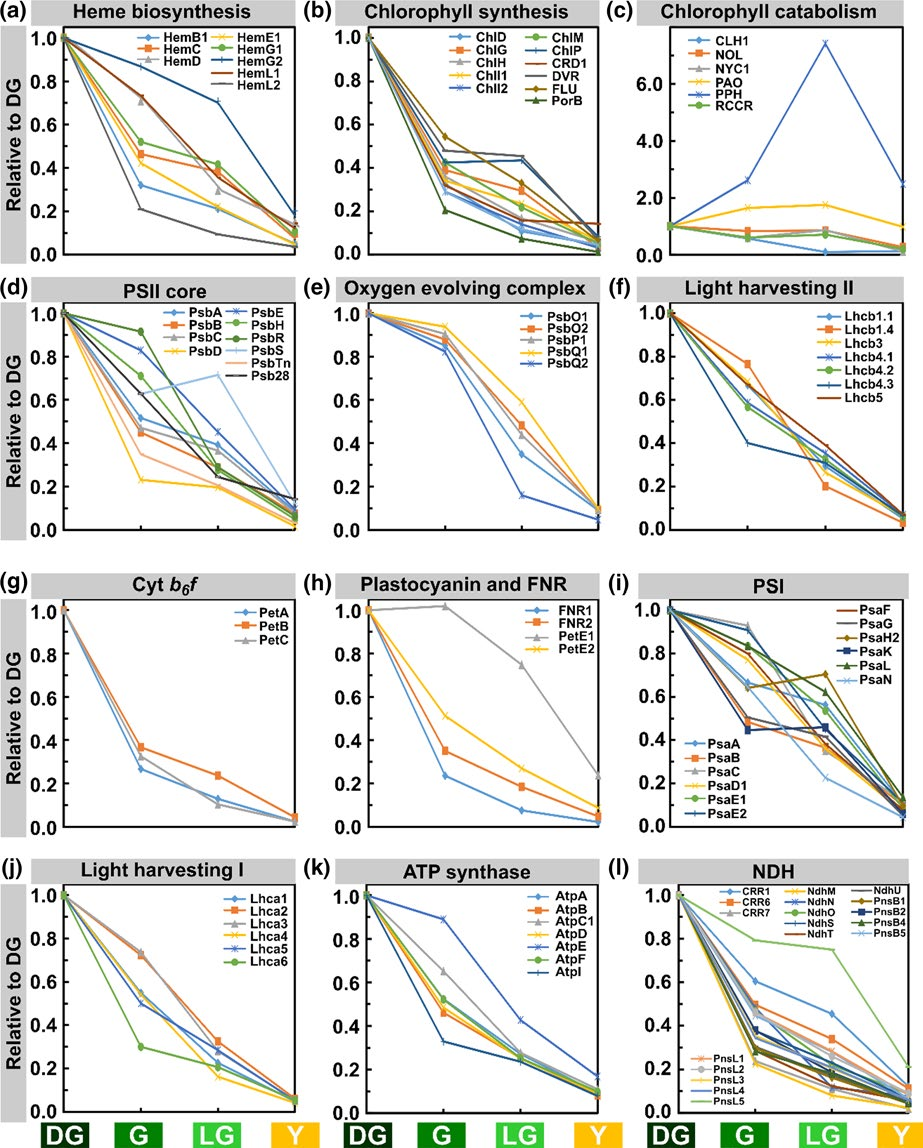
Behavior of proteins in different chloroplast protein groups. Proteomic data derived from the four stages of senescence. Values are based on normalization to leaf area. Proteins were selected from those that passed the ANOVA significance test (*p *<* *0.05). Protein levels are relative to the DG stage

During senescence, the level of enzymes involved in heme and chlorophyll synthesis decreased dramatically, consistent with the drop of chlorophyll levels (Figure 7a,b). CHLP, which catalyzes the reduction in geranyl diphosphate to phytyl diphosphate, and DVR, which catalyzes the conversion of divinyl chlorophyllide to monovinyl chlorophyllide (Meguro, Ito, Takabayashi, Tanaka, & Tanaka, 2011; Tanaka, Oster, Kruse, Rudiger, & Grimm, 1999), remained relatively stable between G and LG stages. The level of enzymes involved in chlorophyll breakdown increased during senescence (Figure 1c), with the exception of the chlorophyllase CLH1, which has been reported not to play a role in leaf senescence in Arabidopsis (Schenk et al., 2007). Levels of all chlorophyll catabolic enzymes, excluding CLH1, decreased in Y leaves compared to LG.

Relative levels of proteins composing PSII also decreased during senescence, but to a lesser extent than of those of enzymes involved in chlorophyll biosynthesis (Figure 7d). PSII proteins exhibited a wide gamut of decreasing patterns. In G leaves, the relative protein levels ranged from 20% to 90% of DG levels. PsbA, B and C, encoding for D1, CP47 and CP43, respectively, act similarly in G leaves and decrease to around 50% of their level in DG. Interestingly, PsbD, the D2 component of PSII reaction center, sharply decreased to about 20% of its initial levels. Levels of inner PSII proteins decreased faster while the more external proteins, such as PsbR, a subunit that stabilizes the binding of PsbP (Suorsa et al., 2006), and PsbS declined by only 10 and 30%, respectively. Proteins of the oxygen evolving complex (OEC) acted alike and, in contrast to PSII core proteins, decreased by only 10% in G compared to DG (Figure 7e). They then dropped in LG, their levels varying but generally still higher than of PSII core proteins.

Light‐harvesting antenna proteins of PSII, which incorporate large amounts of chlorophyll, behaved more uniformly than PSII proteins. Their amounts also decreased in G but only to 60%–‐80% of their initial levels, with the exception of Lhcb4.3 whose level was down to 40% (Figure 7f). In G leaves, the decrease in minor Lhcs (Lhcb4.1‐3 and Lhcb5) was more prominent than the decrease in major Lhcs (Lhcb1.1, Lhcb1.4 and Lhcb3). This trend was reversed in LG, where the decrease in minor Lhcs levels was more subtle than the change observed in levels of major Lhcs.

The three proteins of the cytochrome *b*_*6*_ *f* (Cyt *b*_*6*_ *f*) complex that were detected behaved similarly. PetE2, the predominant form of plastocyanin, decreased by half in G and continued to decrease to 10% in Y (Figure 7h). PetE1, on the other hand, increased slightly in G compared to DG. In LG, its levels dropped but were still at 75% of their initial values. The levels of FNR1 and 2 decreased similar to Cyt *b*_*6*_ *f* proteins and sharply dropped to less than 40% of their DG values, and continued to decrease in LG and Y (Figure 7h).

The behavior of PSI subunits was variable, their levels decreasing in G to values ranging from 95% to 45% of their original ones (Figure 7i). The proteins whose levels decreased the most during the transition were PsaB, which binds the reaction center chlorophyll P700, PsaG, involved in the binding of Lhca1, Lhca4 and plastocyanin, and PsaK, which binds Lhca2 and Lhca3 (Jensen, Rosgaard, Knoetzel, & Scheller, 2002; Jensen et al., 2007; Zygadlo, Jensen, Leister, & Scheller, 2005). On the other hand, the levels of PsaC, which contain two iron sulfur clusters, and of PsaE2, which stabilize the interaction of PsaC with the PSI core, decreased by only 5%. In LG leaves, PSI protein levels continued to decrease with the exception of PsaK and PsaH2, the latter is essential for the docking of LHCII to PSI in state 2 (Lunde, Jensen, Haldrup, Knoetzel, & Scheller, 2000; Varotto et al., 2002). The lowest relative level in LG leaves was of PsaN (Haldrup, Naver, & Scheller, 1999), the only subunit of PSI solely located in the lumen. The amounts of Lhca proteins also decreased significantly upon the transition from DG to G but to various extents, ranging from 30% to 70% of their initial levels (Figure 7j). The levels of Lhca2 and 3 changed similarly and decreased the least out of the Lhcas, to 70% of DG levels. The levels of Lhca1 and Lhca4, which always function as a heterodimer, also changed in a concerted manner, but decreased more pronouncedly to 55% of their DG values. The levels of the minor PSI antenna proteins, Lhca5 and Lhca6, likewise decreased significantly upon transition from DG to G, by 45% and 70%, respectively.

ATP synthase protein, AtpI, an a‐type subunit of CF0, decreased the most upon the DG to G transition, by 70%, while that of the AtpE, the γ subunit of CF1, decreased the least, by only 10%. In Y leaves, the relative levels of AtpE remained slightly higher at around 20%.

The levels of most components of the NDH complex, a PSI‐interacting complex involved in cyclic electron transport (Shikanai, 2016), decreased by 50% or more during the initial stage of senescence, and continued decreasing to about 15% of their initial values in Y leaves (Figure 7l).

### 3.7 Behavior of senescence and autophagy proteins

**Figure 8:**
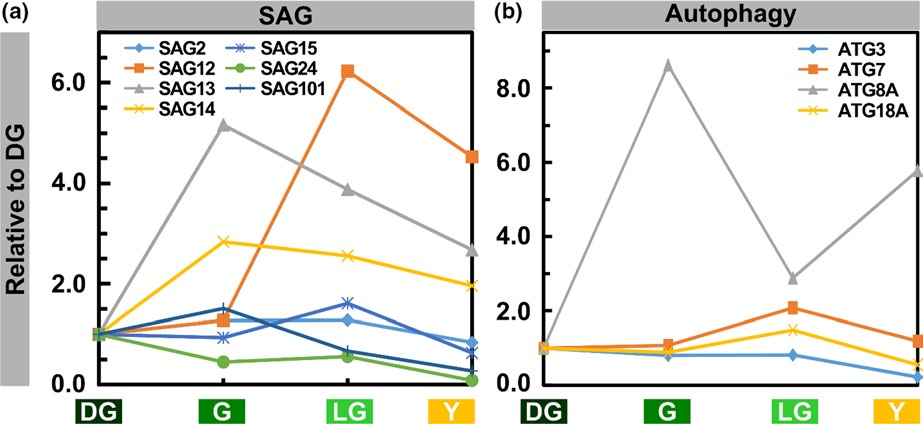
Levels of SAG and Autophagy Proteins During Senescence. (a) SAG proteins. (b) Autophagy proteins. Values are based on proteomic data normalized to leaf area. Proteins were selected from those that passed the ANOVA significance test (*p *<* *0.05). Protein levels are relative to the DG stage

As can be expected, the levels of senescence‐associated gene (SAGs) products increased during senescence. The levels of the SAG13 protein, a short‐chain alcohol dehydrogenase, and SAG14, a blue copper binding protein (Lohman, Gan, John, & Amasino, 1994), notably increased at the G stage by fivefold and threefold, respectively (Figure 8a). In LG and Y, they decreased but were still higher than in DG. The levels of SAG12, a vacuolar cysteine protease, slightly increased in G and dramatically increased in LG by more than sixfold compared to initial values. This is consistent with previous reports referring to SAG12 as a late SAG (Jing, Sturre, Hille, & Dijkwel, 2002; Lohman et al., 1994). In Y, SAG12 levels decreased but were still 4.5 times higher than in DG. Lower levels of upregulation were observed for SAG2—another vacuolar cysteine proteinase, SAG15—a ClpD protease, and the acyl hydrolase SAG101 (He & Gan, 2002; Hensel, Grbic, Baumgarten, & Bleecker, 1993; Lohman et al., 1994).

Four different autophagy proteins, ATG3, ATG7, ATG8A, and ATG18, were detected in our analysis (Figure 8b). ATG3 is an E2 conjugating enzyme and ATG7 is known to activate ATG8 and 12. ATG8A and 18A are involved in autophagosome formation (Doelling, Walker, Friedman, Thompson, & Vierstra, 2002; Michaeli et al., 2014; Xiong, Contento, & Bassham, 2005). All these were upregulated during senescence to various extents. ATG8A levels increased by more than eightfold in G compared to DG, then decreased in LG, and increased again to almost sixfold of its initial value by Y. Levels of ATG7 and 18A peaked in LG, and remained relatively high even in Y leaves. Although ATG3 levels did not increase during senescence, they decreased only slightly through G and LG stages.

### 3.8 Average changes in levels of groups of proteins

**Figure 9:**
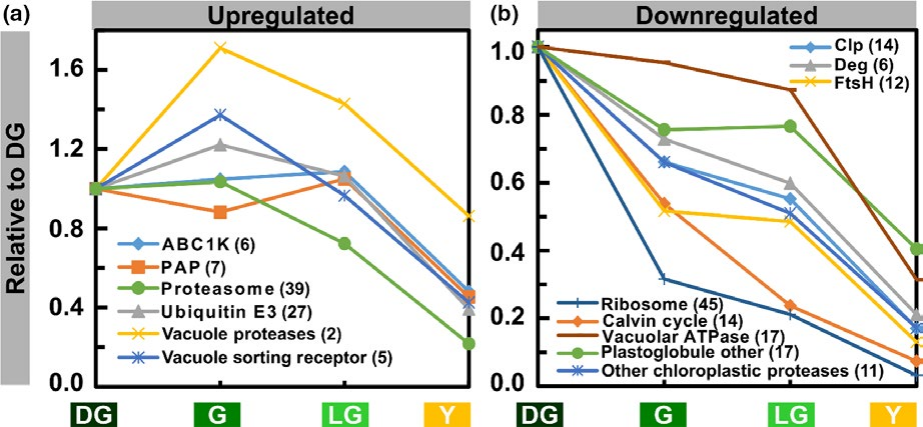
Behavior of Selected Protein Groups During Senescence. (a) Upregulated protein groups. Groups were considered upregulated if their level exceeded their level in a previous stage at least once. (b) Downregulated protein groups. Values are means of levels of proteins comprising a group. Numbers in parentheses indicate the number of proteins in each group. Protein levels are relative to the DG stage

Other protein groups, chloroplastic and nonchloroplastic, were also monitored; their average relative levels are shown Figure 9. They were considered upregulated if their level exceeded at some point the one they have in DG leaves (Figure 9a); if not, they were labeled as downregulated (Figure 9b). Protein synthesis in the chloroplast and the assimilation of carbon dioxide into sugars were two processes that diminished early on during senescence. The level of chloroplast ribosomal proteins decreased sharply by 70% in G compared to DG (Figure 9b), suggesting that protein synthesis capacity in the chloroplast is lost very early upon aging of the leaves. This was not the case with cytosolic ribosomes, whose levels decreased at a much lower rate (Supporting Information Table S1). The levels of Calvin cycle proteins also decreased considerably in G to just above half of their original values, with the exception of GAPA2, that catalyzes the reduction in 1,3‐diphosphoglycerate by NADPH, which increased between DG and G (Supporting Information Table S1). The levels of the cycle's proteins were nearly halved in each of the subsequent stages.

PGs, the thylakoid‐associated lipoprotein particles, whose number and size increased during senescence (Figure 3), contain about 30 proteins (Rottet et al., 2015; van Wijk & Kessler, 2017). Three groups of such proteins were detected in our analysis: ABC1K, a group of six atypical plastid protein kinases; PAP, seven probable plastid‐lipid‐associated proteins; and other PG proteins (17 of them). Level of ABC1Ks slightly increased until the LG stage and only decreased in Y (Figure 9a). One of them, ABC1K7, a senescence‐induced ABC1 KINASE7 (Bhuiyan & van Wijk, 2017), doubled in LG and remained higher than initial levels even in Y leaves (Supporting Information Table S1). The level of almost all PAP proteins slightly decreased in G, increased in LG, and decreased again in Y (Figure 9a). The levels of all other PG proteins never exceeded their initial values (Figure 9b). The level of many of these proteins increased from G to LG and decreased following the transition to Y (Supporting Information Table S1).

Proteins belonging to the major chloroplast protease families Clp, FtsH and Deg, as well as other chloroplastic proteases, were mostly downregulated during senescence, the levels of many of which decreasing by 30%–50% early on following the transition to G (Figure 9b and Supporting Information Table S1). ClpD, also named SAG15, was the only Clp protein whose levels increased during senescence (Supporting Information Table S1). Out of the “Other Chloroplastic Proteases”, SPP, encoding a metalloprotease essential for embryonic development (Trosch & Jarvis, 2011), maintained its initial levels in early stages of senescence but dropped sharply in LG (Supporting Information Table S1). In LG, the levels of all chloroplast protease groups were at 50%–60% of their initial values and decreased to a similar extent in Y (Supporting Information Table S1).

Two other groups of proteins, both residing outside the chloroplast, which bear direct relevance to senescence are vacuole proteins and the ubiquitin 26S‐proteasome system. Vacuolar proteases and vacuole sorting receptors reached their peak levels in G, increasing by 70% and 40%, respectively (Figure 9a). The levels of the former decreased in LG, but were still higher than in DG, and continued to decrease in Y to 90% of their initial values. Following their initial increase, the levels of vacuole sorting receptors steadily decreased throughout the subsequent stages, culminating at about 40% of their initial values in Y. VSR6 (Vacuolar‐sorting receptor 6), involved in sorting proteins from the Golgi apparatus to vacuoles (Wang et al., 2015), a process which is relevant also to autophagy, increased the most, with over a twofold increase in G (Supporting Information Table S1). The level of 17 vacuolar ATPase subunits, which generates the proton gradients used for transport of compounds into this compartment (Gaxiola, Palmgren, & Schumacher, 2007), decreased slightly during G and LG to just under 90% of their initial values, followed by a sharp decrease to 30% in Y (Figure 9a).

Ubiquitin E3s, which attach ubiquitin to target proteins (Mazzucotelli et al., 2006), reached maximum levels at G, following a 20% increase. In LG, they decreased back to DG levels and in Y leaves they dropped to 40% of their initial values. The levels of UBC32, a stress‐induced ER membrane localized ubiquitin conjugation enzyme (Cui et al., 2012), increased by 4.5‐fold in G, continued to increase during the transition to LG, and were still fourfold higher than DG values in Y (Supporting Information Table S1). Proteasome proteins remained at their initial levels in G (Figure 9a). In LG and Y, their levels were lower and were similar to each other at about 70% and 20% of their DG values in LG and Y leaves, correspondingly. The observed relatively high levels of proteasome and ubiquitin‐related proteins, as well as of vacuolar proteins, suggest that protein degradation outside chloroplasts continues well into late stages of senescence.

### 3.9 Degradation of chloroplast proteins during senescence does not correlate with their stability at earlier developmental stages

Our analysis indicates that the disappearance of chloroplast proteins during senescence follows different patterns. As shown in Figure 7, the level of some proteins is maintained relatively high upon the transition from DG to G (like those of the OEC), whereas others disappear faster (like those of the Cyt *b*_*6*_ *f* complex). Similar differences are observed between subunits of the same complex. These differences raise the question of whether the disappearance of proteins during senescence correlates with their degradation rates in leaves during earlier stages of growth and development. In a recent work, Li et al. (2017) determined the degradation rate of hundreds of Arabidopsis proteins. Supporting Information Figure S1 shows the degradation rates for photosynthetic proteins against the ratio of their levels in the G and DG stages. As can be seen, no correlation is apparent suggesting that the rate of disappearance of proteins during senescence is not determined by their inherent stability, as manifested by their degradation rate during earlier developmental stages.

## 4 DISCUSSION

The breakdown of chloroplasts has traditionally been considered as the earliest and most significant structural change occurring during leaf senescence (Lim et al., 2007). A closer look at senescence, as was taken here, reveals that not all processes associated with chloroplast breakdown proceed simultaneously. Using chlorophyll level as a marker for defining distinct stages in leaf senescence, we demonstrate that chlorophyll catabolism precedes all other structural changes, as well as changes in cellular and organellar proteomes and, likely, in their corresponding metabolomes. At each one of the senescence stages examined here, the loss of chlorophyll was more pronounced than the loss of protein (Figure 1). Moreover, a decrease in chloroplast volume was not observed before the LG stage, and the volume chloroplasts occupied out of the cell volume significantly decreased only at LG and Y stages. At the ultrastructural level, thylakoids retained their normal appearance till the last stage of senescence. The only exception was PGs, whose size and number continuously increased throughout senescence, beginning well before clear changes in thylakoid structure and organization can be observed. This description refines earlier notions of antiparallel changes in thylakoid membranes morphology and PGs number and size (Lim et al., 2007; Nooden, 1988).

As the level of chlorophyll at each developmental stage represents a balance between synthesis and degradation, its continuous decrease could result from either a decrease in synthesis, increase in degradation, or both. In the absence of data on chlorophyll synthesis and degradation rates during the different stages, these can be only indirectly inferred from the levels of enzymes involved in these processes. HemA, which catalyzes the important step of ALA synthesis in the chlorophyll biosynthesis pathway (Ilag, Kumar, & Soll, 1994), and SGR, which catalyzes the first committed step in the chlorophyll degradation pathway (Shimoda, Ito, & Tanaka, 2016), were not detected here. From the results of the remaining proteins presented in Figure 7a–c, however, a decrease in biosynthesis may also contribute to the decrease in the level of chlorophyll during senescence. Upon transition from DG to G, the level of enzymes involved in chlorophyll biosynthesis decreases by 50%–80%, whereas the level of those involved in its breakdown does not change much, with the exception of PPH whose level increases by more than twofold during the transition from DG to G.

The observation that chlorophyll degradation is the earliest noticeable event during leaf senescence suggests that it enables or signals further processes in the chloroplast. This possibility is supported by the phenotypes of Stay‐Green mutants that are impaired in chlorophyll catabolism, such as the *nyc1*,* pph‐1* and *Sgr* mutants (Kusaba et al., 2007; Park et al., 2007; Schelbert et al., 2009). In these mutants, thylakoid membranes and the level of major LHCII proteins are retained longer than in WT plants, whereas other proteins are degraded.

Accumulation of chlorophyll‐binding proteins during chloroplast biogenesis is dependent on chlorophyll availability. In the absence of chlorophyll, such proteins are synthesized but rapidly degraded (reviewed in (Adam, 1996). This tight correlation between accumulation of chlorophyll and chlorophyll‐binding proteins does not appear to hold during senescence. In stage G, after more than 50% of the chlorophyll has been degraded, the level of the major LHCIIs, Lhcb1.1, Lhcb1.4, and Lhcb3, drops by only 20%–40% (whereas PSI and PSII core proteins drop significantly more). This suggests that many chlorophyll‐binding proteins do not possess a full complement of their pigments, and yet they are relatively stable. Obviously, this does not imply that they are active in capturing light energy, but it suggests that their degradation is not tightly coupled to the loss of their cofactors and activity. Although the pathway of chlorophyll catabolism during senescence is now fully established (Kuai et al., 2018), how the pigments are released from the hydrophobic pockets within their binding proteins, to become accessible to the catabolizing enzymes, is far from being understood. One can nevertheless assume that once chlorophyll‐binding proteins lose their pigments, their conformation loosens, making them more susceptible to proteolysis.

Aside from their essential role in capturing light energy, LHCII proteins have been implicated in initiating and maintaining membrane stacking in the appressed (granal) domains of the thylakoid network (Day, Ryrie, & Fuad, 1984; Goodchild, Highkin, & Boardman, 1966; Nevo, Charuvi, Tsabari, & Reich, 2012). Although chlorophyll b is important for the stability of LHCs, in mutant Arabidopsis plants lacking chlorophyll b some level of thylakoid stacking is retained (Kim et al., 2009; Murray & Kohorn, 1991). It is still surprising to observe that during senescence, the relation between the level of LHCIIs and thylakoid membrane stacking is not very tight. At the LG stage, after >70% of chlorophyll and 60%–80% of LHCII proteins have already been degraded, the stacking of thylakoid membranes in the grana appears perfectly normal. Whether these are the remaining LHCIIs or other membrane constituents that are sufficient to retain stacking is still unknown.

Chlorophyll levels, and to less extent levels of various PSII subunits, decrease during the transition from DG to LG (Figures 1A and 7d,e). Concomitantly, the Fv/Fm ratio remains stable (Figure 1c). This means that although fewer PSII complexes are present in the leaves as senescence advances, the remaining complexes function with the same efficiency. It seems that PSII complexes are degraded in a sequential manner rather than all in parallel, enabling the remaining PSII complexes to keep their normal efficiency until advanced stages of senescence. Only in the final stage of senescence the PSII efficiency drops noticeably.

Although the level of the great majority of chloroplast proteins decreases during senescence, the patterns of their disappearance are quite variable. The level of many proteins starts declining upon transition from DG to G stage, but the level of a considerable number of proteins begins to decline later on, upon transition from G to LG. No apparent rule seems to govern this differential behavior. Furthermore, sometimes the behavior of components of the same protein complex can be different, e.g., subunits of PSII and PSI. The lack of correlation between the changes in the levels of photosynthetic proteins upon transition from DG to G with their reported stabilities during early developmental stages further highlights the complexity of protein degradation during senescence.

The primary contributor to the declining levels of proteins is probably the diminution of the chloroplast protein translation machinery, resulting from the sharp decrease in the level of chloroplastic ribosomal proteins. Upon transition from DG to G, almost 70% of these proteins are lost (Figure 9). By comparison, only 40% of cytosolic ribosomal proteins disappear during this transition. The second effector is proteases. Chloroplasts contain a large arsenal of proteases which are essential for the homeostasis of the organelle. Of these, the one that is most convincingly linked to senescence is the PG‐associated metalloprotease PGM48 (Bhuiyan & van Wijk, 2017). Although it positively regulates senescence, its levels decrease to ca. 70%, 50% and 15% of its DG level in G, LG and Y stages, respectively. A similar decrease is observed for almost all other chloroplast proteases, including stromal Clp protease and thylakoid FtsH and Deg proteases. As they are all held at 50%–60% of their DG levels as late as the LG stage (Figure 9 and Supporting Information Table S1), they are likely to participate in degradation of chloroplast proteins during senescence. However, the involvement of specific proteases in this process still awaits demonstration.

Degradation of whole chloroplasts by chlorophagy has been documented for dark‐induced senescence and following oxidative stress (Izumi et al., 2017; Nakamura & Izumi, 2018; Wada et al., 2009). However, evidence for whole chloroplast engulfment during natural senescence is still missing. It has been previously suggested that chlorophagy should not play a role in chloroplast dismantling during senescence because autophagy mutants demonstrate early— rather than late—senescence phenotype, compared with WT (Doelling et al., 2002; Otegui, 2018; Thompson, Doelling, Suttangkakul, & Vierstra, 2005). Consistent with this suggestion, we observed neither intact chloroplasts nor large chunks of thylakoids within vacuoles in our EM images. Nevertheless, since the autophagy‐related proteins ATG3, ATG7, ATG8A and ATG18A are maintained at relatively high levels throughout senescence, as are vacuolar ATPases and proteases (Figures 8 and 9), other autophagy processes, including mitophagy, may continue even in late stages of senescence and contribute their share to remobilization of resources to other parts of the plant.

## 5 CONCLUSION

**Figure 10:**
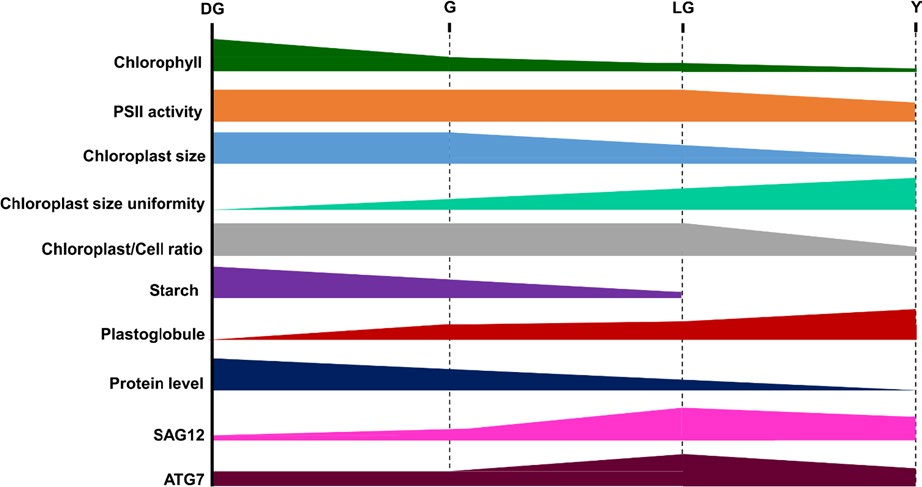
Characteristics of senescence. A schematic representation of the behavior of different parameters throughout four stages of senescence

The observations made in this study are summarized in Figure 10, to draw a comparative picture of different cellular and organellar events occurring during senescence. As natural leaf senescence proceeds, leaves continuously lose their chlorophyll and proteins, although the loss of the former precedes all other processes. Chloroplast size starts decreasing only after the G stage, and the distribution of sizes becomes progressively more and more uniform throughout senescence. PSII potential remains relatively high until late stages, but starch reserves are rapidly declining until the LG stage, indicating impaired capacity for carbon fixation. Thylakoid membranes ultrastructure and organization appear normal until the last stage of senescence, unlike the number and size of PGs, which increase continuously throughout the process. These highlight chlorophyll catabolism as the process that precedes all other changes, and may enable or signal the advancement of all other processes leading to the leaf's death.

## CONFLICT OF INTEREST

The authors declare no conflict of interest associated with the work described in this manuscript.

## AUTHORS CONTRIBUTIONS

E.T., Y.E., Z.R. and Z.A. designed the experimental work. E.T., R.N., S.L.‐Z. and V.K. performed all the microscopy work. M.K. and Y.L. carried out the MS analysis. All other aspects of the experimental work were carried out by E.T. L.N. All data were analyzed by E.T., Y.E., Z.R. and Z.A. E.T. and Z.A. wrote the article. Z.A. supervised the project.
