# Supplementary material for: Chlorophyll catabolism precedes changes in chloroplast structure and proteome during leaf senescence
Source: Plant Direct. 2019 Mar 20;3(3):e00127. doi: 10.1002/pld3.127 (PMC6508775; doi:10.1002/pld3.127)
Supplement: Supplementary file 1 [file PLD3-3-e00127-s001.pdf]

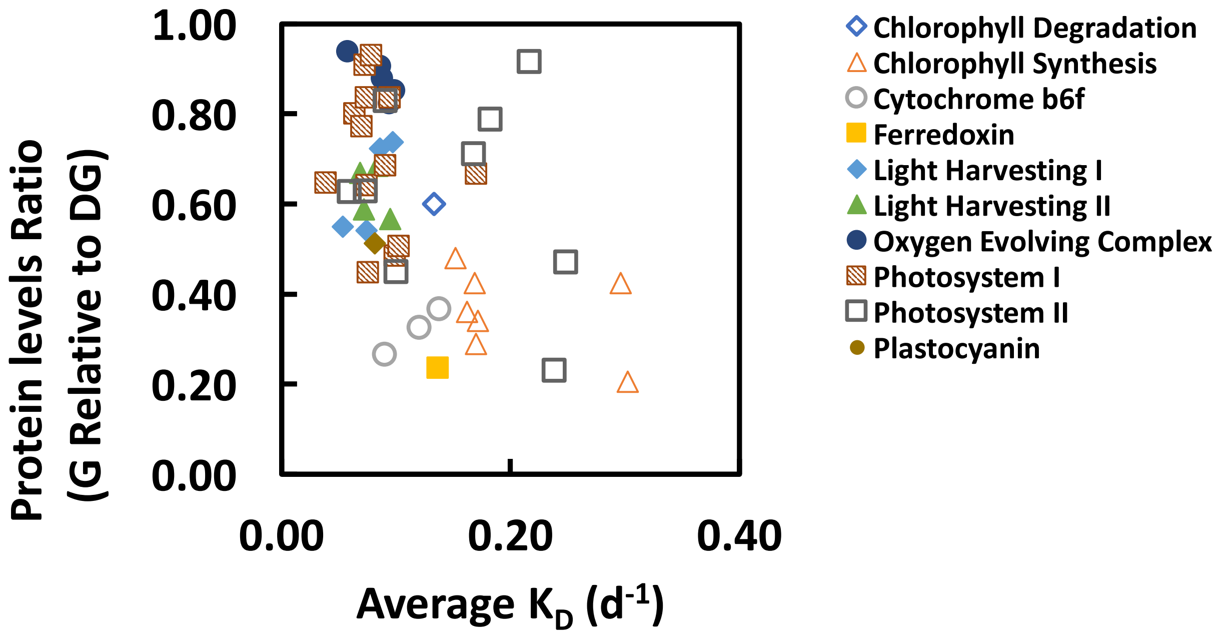

Fig. S1 - Correlation between degradation rates of chloroplast proteins in mature and senescing leaves.

Y axis - Levels of *Arabidopsis thaliana* chloroplast proteins (shown in Fig. 7) in Green (G) stage, compared to their levels in Dark Green (DG). X axis – Degradation rates of these proteins as determined by Li et al. 2017.
