# Supplementary material for: Chlorophyll catabolism precedes changes in chloroplast structure and proteome during leaf senescence
Source: Plant Direct. 2019 Mar 20;3(3):e00127. doi: 10.1002/pld3.127 (PMC6508775; doi:10.1002/pld3.127)
Supplement: Supplementary file 2 [file PLD3-3-e00127-s002.pdf]

Protein Group	Gene name (TAIR)	Area DG Relative to DG	Area G Relative to DG	Area LG Relative to DG	Area Y Relative to DG
ABC1K	ABC1K1	1.00	0.74	0.82	0.33
ABC1K	ABC1K3	1.00	1.17	1.49	0.56
ABC1K	ABC1K5	1.00	0.67	0.65	0.27
ABC1K	ABC1K6	1.00	0.71	0.47	0.18
ABC1K	ABC1K7	1.00	1.82	2.14	1.13
ABC1K	ABC1K9	1.00	1.17	0.96	0.41
ABC1K	Average	1.00	1.05	1.09	0.48
Calvin cycle	CFBP1	1.00	0.31	0.10	0.04
Calvin cycle	CP12-1	1.00	0.68	0.60	0.19
Calvin cycle	CP12-2	1.00	0.40	0.23	0.04
Calvin cycle	GAPA1	1.00	0.38	0.27	0.05
Calvin cycle	GAPA2	1.00	1.38	0.23	0.21
Calvin cycle	PRK	1.00	0.50	0.24	0.06
Calvin cycle	RBCL	1.00	0.63	0.27	0.07
Calvin cycle	RBCS-1A	1.00	0.56	0.21	0.06
Calvin cycle	RBCS-1B	1.00	0.56	0.25	0.05
Calvin cycle	RBCS-2B	1.00	0.48	0.23	0.05
Calvin cycle	RCA	1.00	0.34	0.12	0.03
Calvin cycle	SBPASE	1.00	0.28	0.12	0.04
Calvin cycle	TIM	1.00	0.48	0.26	0.07
Calvin cycle	TKL-1	1.00	0.52	0.19	0.08
Calvin cycle	Avergae	1.00	0.54	0.24	0.07
Clp	ClpB3	1.00	0.53	0.35	0.15
Clp	ClpC1	1.00	0.70	0.54	0.16
Clp	ClpD	1.00	0.93	1.61	0.63
Clp	ClpF	1.00	0.37	0.18	0.05
Clp	ClpP1	1.00	0.65	0.51	0.15
Clp	ClpP3	1.00	0.68	0.44	0.12
Clp	ClpP4	1.00	0.56	0.28	0.07
Clp	ClpP5	1.00	0.59	0.40	0.07
Clp	ClpP6	1.00	0.59	0.52	0.12
Clp	ClpR1	1.00	0.83	0.77	0.24
Clp	ClpR2	1.00	0.75	0.48	0.14
Clp	ClpR3	1.00	0.63	0.43	0.16
Clp	ClpR4	1.00	0.65	0.51	0.18
Clp	ClpT1	1.00	0.83	0.72	0.17
Clp	ClpT2	1.00	0.62	0.54	0.12
Clp	Average	1.00	0.66	0.55	0.17
Deg	DegP1	1.00	0.78	0.55	0.21
Deg	DegP2	1.00	0.50	0.37	0.08
Deg	DegP5	1.00	0.71	0.57	0.25
Deg	DegP7	1.00	1.02	0.88	0.35
Deg	DegP8	1.00	0.56	0.59	0.19
Deg	DegP9	1.00	0.81	0.62	0.18
Deg	Average	1.00	0.73	0.60	0.21
FtsH	FtsH1	1.00	0.75	0.43	0.18
FtsH	FtsH11	1.00	0.68	0.42	0.12
FtsH	FtsH12	1.00	0.51	0.46	0.12
FtsH	FtsH2	1.00	0.49	0.29	0.13
FtsH	FtsH5	1.00	0.51	0.36	0.11
FtsH	FtsH8	1.00	0.62	0.60	0.23
FtsH	FtsH9	1.00	0.46	0.17	0.06
FtsH	FtsHI1	1.00	0.39	0.32	0.08
FtsH	FtsHI2	1.00	0.69	0.71	0.18
FtsH	FtsHI3	1.00	0.29	0.27	0.08
FtsH	FtsHI4	1.00	0.27	1.14	0.09

FtsH	FtsHI5	1.00	0.54	0.65	0.18
FtsH	Average	1.00	0.52	0.49	0.13
Other chloroplastic proteases	ARASP	1.00	0.41	0.80	0.26
Other chloroplastic proteases	ARASP2	1.00	0.53	0.45	0.15
Other chloroplastic proteases	EGY1	1.00	0.34	0.15	0.06
Other chloroplastic proteases	EGY2	1.00	0.75	0.74	0.25
Other chloroplastic proteases	LON1	1.00	0.76	0.59	0.16
Other chloroplastic proteases	OOP	1.00	0.87	0.65	0.24
Other chloroplastic proteases	PGM48	1.00	0.69	0.51	0.15
Other chloroplastic proteases	PLSP1	1.00	0.49	0.37	0.08
Other chloroplastic proteases	PREP1	1.00	0.66	0.50	0.15
Other chloroplastic proteases	SPP	1.00	1.06	0.20	0.08
Other chloroplastic proteases	SPPA	1.00	0.71	0.64	0.31
Other chloroplastic proteases	Average	1.00	0.66	0.51	0.17
PAP	PAP1	1.00	0.62	0.49	0.22
PAP	PAP10	1.00	0.81	0.91	0.28
PAP	PAP13	1.00	0.72	0.85	0.26
PAP	PAP2	1.00	1.63	2.81	1.40
PAP	PAP3	1.00	0.81	0.82	0.37
PAP	PAP6	1.00	0.96	0.82	0.42
PAP	PAP9	1.00	0.62	0.64	0.23
PAP	Average	1.00	0.88	1.05	0.45
Plastoglobule other	AKR	1.00	0.45	0.28	0.08
Plastoglobule other	At3g43540	1.00	0.97	0.52	0.05
Plastoglobule other	At4g13200	1.00	0.84	1.24	0.61
Plastoglobule other	CCD4	1.00	0.35	0.26	0.03
Plastoglobule other	Esterase 1	1.00	0.64	0.73	0.11
Plastoglobule other	FVR1	1.00	0.69	0.53	0.21
Plastoglobule other	FVR2	1.00	0.60	0.45	0.21
Plastoglobule other	HBP3	1.00	0.89	0.73	0.26
Plastoglobule other	NDC1	1.00	0.67	0.71	0.22
Plastoglobule other	PES1	1.00	0.89	1.80	1.80
Plastoglobule other	PES2	1.00	1.18	1.33	0.53
Plastoglobule other	PGM48	1.00	0.69	0.51	0.15
Plastoglobule other	PGSAG	1.00	1.03	0.90	0.30
Plastoglobule other	PLAT1	1.00	0.89	0.95	0.37
Plastoglobule other	UBIE-MT1	1.00	0.62	0.55	0.21
Plastoglobule other	UBIE-MT2	1.00	0.70	0.60	0.27
Plastoglobule other	VTE1	1.00	0.82	0.72	0.49
Plastoglobule other	Average	1.00	0.76	0.75	0.35
Proteasome	PAA1	1.00	0.83	0.54	0.16
Proteasome	PAA2	1.00	1.46	0.93	0.36
Proteasome	PAB1	1.00	1.16	0.52	0.25
Proteasome	PAB2	1.00	0.78	0.28	0.10
Proteasome	PAC1	1.00	0.99	0.67	0.22
Proteasome	PAD2	1.00	1.26	0.72	0.20
Proteasome	PAE1	1.00	1.00	0.48	0.12
Proteasome	PAF1	1.00	0.97	0.78	0.26
Proteasome	PAG1	1.00	0.92	0.82	0.24
Proteasome	PBA1	1.00	0.97	0.84	0.26
Proteasome	PBB1	1.00	1.11	0.88	0.25
Proteasome	PBB2	1.00	0.82	0.69	0.20
Proteasome	PBC1	1.00	0.68	0.62	0.18
Proteasome	PBC2	1.00	1.87	0.59	0.02
Proteasome	PBD1	1.00	0.77	0.73	0.19
Proteasome	PBD2	1.00	2.39	1.67	0.38
Proteasome	PBE1	1.00	0.82	0.71	0.20
Proteasome	PBE2	1.00	0.71	0.69	0.17

Proteasome	PBG1	1.00	1.04	0.67	0.18
Proteasome	PRC6A	1.00	1.93	0.64	0.22
Proteasome	RPN10	1.00	0.90	0.61	0.10
Proteasome	RPN11	1.00	0.87	0.35	0.09
Proteasome	RPN12A	1.00	0.85	0.65	0.18
Proteasome	RPN1A	1.00	0.73	0.97	0.33
Proteasome	RPN1B	1.00	1.14	1.06	0.39
Proteasome	RPN2A	1.00	0.88	0.64	0.15
Proteasome	RPN2B	1.00	1.21	0.89	0.31
Proteasome	RPN3B	1.00	0.66	0.40	0.15
Proteasome	RPN6	1.00	1.03	0.98	0.28
Proteasome	RPN7	1.00	0.84	0.62	0.24
Proteasome	RPN8A	1.00	0.98	0.73	0.23
Proteasome	RPN9A	1.00	0.64	0.73	0.15
Proteasome	RPN9B	1.00	0.98	0.88	0.27
Proteasome	RPT1A	1.00	0.91	0.69	0.31
Proteasome	RPT2B	1.00	1.01	0.32	0.20
Proteasome	RPT3	1.00	0.99	0.49	0.18
Proteasome	RPT4B	1.00	1.57	1.52	0.46
Proteasome	RPT5A	1.00	0.92	0.83	0.22
Proteasome	RPT6A	1.00	0.74	0.37	0.09
Proteasome	Average	1.00	1.03	0.72	0.22
Ribosome (Chloroplast)	RPL1	1.00	0.44	0.23	0.03
Ribosome (Chloroplast)	RPL10	1.00	0.33	0.25	0.04
Ribosome (Chloroplast)	RPL11	1.00	0.39	0.28	0.03
Ribosome (Chloroplast)	RPL12A	1.00	0.40	0.29	0.04
Ribosome (Chloroplast)	RPL13	1.00	0.27	0.10	0.02
Ribosome (Chloroplast)	RPL14	1.00	0.34	0.23	0.03
Ribosome (Chloroplast)	RPL15	1.00	0.28	0.21	0.02
Ribosome (Chloroplast)	RPL16	1.00	0.28	0.18	0.02
Ribosome (Chloroplast)	RPL17	1.00	0.37	0.25	0.05
Ribosome (Chloroplast)	RPL18	1.00	0.63	0.12	0.05
Ribosome (Chloroplast)	RPL2	1.00	0.25	0.24	0.02
Ribosome (Chloroplast)	RPL20	1.00	0.20	0.18	0.02
Ribosome (Chloroplast)	RPL21	1.00	0.39	0.30	0.04
Ribosome (Chloroplast)	RPL22	1.00	0.32	0.25	0.03
Ribosome (Chloroplast)	RPL23	1.00	0.29	0.22	0.03
Ribosome (Chloroplast)	RPL24	1.00	0.26	0.17	0.02
Ribosome (Chloroplast)	RPL27	1.00	0.24	0.23	0.03
Ribosome (Chloroplast)	RPL28	1.00	0.21	0.15	0.02
Ribosome (Chloroplast)	RPL29	1.00	0.31	0.28	0.03
Ribosome (Chloroplast)	RPL31	1.00	0.32	0.20	0.03
Ribosome (Chloroplast)	RPL3A	1.00	0.36	0.21	0.04
Ribosome (Chloroplast)	RPL4	1.00	0.37	0.24	0.05
Ribosome (Chloroplast)	RPL5	1.00	0.29	0.21	0.02
Ribosome (Chloroplast)	RPL6	1.00	0.45	0.20	0.05
Ribosome (Chloroplast)	RPL9	1.00	0.28	0.21	0.02
Ribosome (Chloroplast)	RPS1	1.00	0.37	0.21	0.03
Ribosome (Chloroplast)	RPS10	1.00	0.30	0.15	0.03
Ribosome (Chloroplast)	RPS11	1.00	0.27	0.19	0.04
Ribosome (Chloroplast)	RPS12	1.00	0.23	0.04	0.02
Ribosome (Chloroplast)	RPS13	1.00	0.39	0.31	0.04
Ribosome (Chloroplast)	RPS14	1.00	0.20	0.11	0.02
Ribosome (Chloroplast)	RPS15	1.00	0.18	0.14	0.01
Ribosome (Chloroplast)	RPS16-1	1.00	0.38	0.14	0.03
Ribosome (Chloroplast)	RPS17	1.00	0.33	0.26	0.04
Ribosome (Chloroplast)	RPS18	1.00	0.20	0.22	0.03
Ribosome (Chloroplast)	RPS19	1.00	0.32	0.22	0.04

Ribosome (Chloroplast)	RPS2	1.00	0.36	0.29	0.03
Ribosome (Chloroplast)	RPS20	1.00	0.33	0.24	0.03
Ribosome (Chloroplast)	RPS3	1.00	0.27	0.18	0.03
Ribosome (Chloroplast)	RPS4	1.00	0.23	0.23	0.02
Ribosome (Chloroplast)	RPS5	1.00	0.29	0.25	0.05
Ribosome (Chloroplast)	RPS6	1.00	0.32	0.23	0.03
Ribosome (Chloroplast)	RPS7	1.00	0.29	0.23	0.03
Ribosome (Chloroplast)	RPS8	1.00	0.29	0.23	0.04
Ribosome (Chloroplast)	RPS9	1.00	0.40	0.25	0.04
Ribosome (Chloroplast)	Average	1.00	0.32	0.21	0.03
Ribosome (Cytosol)	RPL13D	1.00	0.62	0.68	0.13
Ribosome (Cytosol)	RPL18AB	1.00	0.37	0.56	0.09
Ribosome (Cytosol)	RPL26B	1.00	0.27	0.37	0.09
Ribosome (Cytosol)	RPL27AC	1.00	0.43	0.50	0.08
Ribosome (Cytosol)	RPL27B	1.00	15.07	10.36	2.75
Ribosome (Cytosol)	RPL27C	1.00	0.59	0.78	0.17
Ribosome (Cytosol)	RPL30A	1.00	0.98	0.68	0.28
Ribosome (Cytosol)	RPL30B	1.00	0.36	0.59	0.16
Ribosome (Cytosol)	RPL32A	1.00	0.58	0.67	0.15
Ribosome (Cytosol)	RPL34B	1.00	0.32	0.19	0.06
Ribosome (Cytosol)	RPL35AD	1.00	0.48	0.47	0.08
Ribosome (Cytosol)	RPL35B	1.00	0.68	0.66	0.11
Ribosome (Cytosol)	RPL36C	1.00	0.26	0.42	0.05
Ribosome (Cytosol)	RPL37AC	1.00	0.25	0.34	0.04
Ribosome (Cytosol)	RPL37B	1.00	0.37	0.33	0.08
Ribosome (Cytosol)	RPL38	1.00	1.12	0.62	0.15
Ribosome (Cytosol)	RPL7A	1.00	0.45	0.24	0.02
Ribosome (Cytosol)	RPP1A	1.00	1.60	1.38	0.24
Ribosome (Cytosol)	RPP1C	1.00	0.67	0.69	0.15
Ribosome (Cytosol)	RPP2A	1.00	0.84	0.26	0.04
Ribosome (Cytosol)	RPP2C	1.00	0.83	1.03	0.50
Ribosome (Cytosol)	RPP3B	1.00	0.44	0.51	0.12
Ribosome (Cytosol)	RPS10A	1.00	0.46	0.54	0.06
Ribosome (Cytosol)	RPS11C	1.00	0.65	0.66	0.14
Ribosome (Cytosol)	RPS15A	1.00	0.70	0.43	0.09
Ribosome (Cytosol)	RPS17B	1.00	0.29	0.07	0.01
Ribosome (Cytosol)	RPS25D	1.00	0.30	0.13	0.04
Ribosome (Cytosol)	RPS25E	1.00	0.39	0.23	0.04
Ribosome (Cytosol)	RPS27AB	1.00	0.95	0.97	0.34
Ribosome (Cytosol)	RPS29	1.00	0.61	0.38	0.08
Ribosome (Cytosol)	RPS30	1.00	0.44	0.35	0.03
Ribosome (Cytosol)	Average*	1.00	0.58	0.52	0.12
Ubiquitin E3	BPM4	1.00	0.79	0.81	0.13
Ubiquitin E3	CAND1	1.00	0.92	0.84	0.25
Ubiquitin E3	CSN3	1.00	1.04	0.65	0.22
Ubiquitin E3	CSN6A	1.00	1.34	0.60	0.22
Ubiquitin E3	CUL3A	1.00	1.14	0.99	0.31
Ubiquitin E3	CUL4	1.00	0.86	0.61	0.28
Ubiquitin E3	DCAF1	1.00	0.94	0.89	0.14
Ubiquitin E3	DDB1A	1.00	3.76	0.25	1.05
Ubiquitin E3	HRD3A	1.00	0.82	0.36	0.14
Ubiquitin E3	KEG	1.00	1.21	1.39	0.47
Ubiquitin E3	PRT6	1.00	1.82	1.70	0.56
Ubiquitin E3	PUB13	1.00	1.15	0.82	0.29
Ubiquitin E3	RGLG1	1.00	1.09	1.60	0.34
Ubiquitin E3	RGLG4	1.00	1.53	0.40	0.29
Ubiquitin E3	RPT2	1.00	0.65	0.53	0.09
Ubiquitin E3	SAUL1	1.00	0.78	0.57	0.12

Ubiquitin E3	SKP1B	1.00	1.14	0.79	0.21
Ubiquitin E3	UBA1	1.00	0.85	5.94	0.32
Ubiquitin E3	UBA2	1.00	0.52	0.34	0.10
Ubiquitin E3	UBC27	1.00	1.36	0.72	0.36
Ubiquitin E3	UBC32	1.00	4.72	6.43	4.06
Ubiquitin E3	UIP1	1.00	0.99	0.66	0.21
Ubiquitin E3	UPL1	1.00	1.03	0.70	0.25
Ubiquitin E3	UPL2	1.00	1.29	1.08	0.31
Ubiquitin E3	UPL3	1.00	0.67	0.38	0.10
Ubiquitin E3	UPL4	1.00	3.18	2.10	0.66
Ubiquitin E3	UPL6	1.00	0.83	0.42	0.17
Ubiquitin E3	Average	1.00	1.22	1.06	0.39
Vacuolar ATPase	AT3G28710	1.00	0.78	0.30	0.45
Vacuolar ATPase	AT3G28715	1.00	1.21	1.22	0.47
Vacuolar ATPase	AT3G42050	1.00	1.16	1.36	0.36
Vacuolar ATPase	AT3G58730	1.00	0.89	0.55	0.28
Vacuolar ATPase	VAB2	1.00	0.95	0.93	0.36
Vacuolar ATPase	VHA-A	1.00	1.08	1.01	0.35
Vacuolar ATPase	VHA-A1	1.00	1.01	0.95	0.33
Vacuolar ATPase	VHA-a2	1.00	0.51	0.75	0.25
Vacuolar ATPase	VHA-A3	1.00	1.05	1.32	0.46
Vacuolar ATPase	VHA-B1	1.00	0.93	0.77	0.17
Vacuolar ATPase	VHA-C	1.00	1.09	1.00	0.35
Vacuolar ATPase	VHA-C1	1.00	0.99	0.75	0.38
Vacuolar ATPase	VHA-E1	1.00	0.91	0.74	0.27
Vacuolar ATPase	VHA-E3	1.00	0.78	0.80	0.15
Vacuolar ATPase	VHA-F	1.00	0.66	0.36	0.19
Vacuolar ATPase	VHA-G1	1.00	0.82	0.72	0.24
Vacuolar ATPase	VHA-G2	1.00	1.38	1.32	0.27
Vacuolar ATPase	Average	1.00	0.95	0.87	0.31
Vacuole proteases	GAMMA-VPE	1.00	1.81	1.48	0.61
Vacuole proteases	RD21	1.00	1.60	1.38	1.11
Vacuole proteases	Average	1.00	1.71	1.43	0.86
Vacuole sorting receptors	VSR1	1.00	1.03	0.91	0.33
Vacuole sorting receptors	VSR3	1.00	0.84	0.73	0.29
Vacuole sorting receptors	VSR4	1.00	1.29	1.36	0.41
Vacuole sorting receptors	VSR6	1.00	2.22	0.87	0.37
Vacuole sorting receptors	VSR7	1.00	1.49	0.96	0.73
Vacuole sorting receptors	Average	1.00	1.37	0.97	0.43

Table S1. Changes in the level of different proteins during senescence.
