# Supplementary material for: Chlorophyll catabolism precedes changes in chloroplast structure and proteome during leaf senescence
Source: Plant Direct. 2019 Mar 20;3(3):e00127. doi: 10.1002/pld3.127 (PMC6508775; doi:10.1002/pld3.127)
Supplement: Supplementary file 4 [file PLD3-3-e00127-s004.docx]

**Reviewer’s comment:**

This reviewer considers that without results of HemA and SGR, it would be difficult to discuss on the synthesis and degradation. The revised sentence "From the results of the remaining proteins ....is" still strong and there is a gap from the preceding sentence. My suggestion is "From the results of the remaining proteins presented in Fig. 7A-C, however, a decrease in biosynthesis may also contribute to the decrease in the level of chlorophyll during senescence." Furthermore, the citation (Sakuraba et al., 2012) is not appropriate because SGR is not the enzyme in that paper. Shimoda (Plant Cell, 2016) would be more appropriate.

**Authors’ response:**

The reviewer’s suggestion is perfectly fine. As for the citation, it was indeed a mistake. In accordance with these two comments, the revised sentences are as follows (lines 450-454 in the highlighted file):

“… and SGR, which catalyzes the first committed step in the chlorophyll degradation pathway (Shimoda et al., 2016), were not detected here. From the results of the remaining proteins presented in Fig. 7A-C, however, a decrease in biosynthesis may also contribute to the decrease in the level of chlorophyll during senescence”.
